# MARCO+ Tumor‐Associated Macrophages Impede CD8+ T Cell Immunity to Facilitate Immunotherapy Resistance in Renal Cell Carcinoma

**DOI:** 10.1002/advs.202514600

**Published:** 2025-10-21

**Authors:** Jiayuan Chen, Jiazhi Mo, Jinchang Wei, Mengnan Qu, Jie Dai, Yan Kong, Huayan Xu, Juan Li, Xieqiao Yan, Chuanliang Cui, Lu Si, Zhihong Chi, Jun Guo, Xiaowen Wu, Xinan Sheng

**Affiliations:** ^1^ Department of Genitourinary Oncology Key Laboratory of Carcinogenesis and Translational Research (Ministry of Education/Beijing) Peking University Cancer Hospital & Institute Peking University Beijing 100142 China; ^2^ Department of Melanoma and Sarcoma Key Laboratory of Carcinogenesis and Translational Research (Ministry of Education/Beijing) Peking University Cancer Hospital & Institute Peking University Beijing 100142 China

**Keywords:** immunotherapy, macrophages, MARCO, renal cell carcinoma, single‐cell RNA‐seq

## Abstract

Immune checkpoint blockade (ICB) therapy, especially in combination regimens, has significantly increased survival of renal cell carcinoma (RCC) patients. However, the ICB‐resistant mechanisms remain largely unclear and require further investigation. Here, an immunosuppressive ecosystem in ICB‐resistant tumors is identified, featured by preferential infiltration of MARCO+ tumor‐associated macrophages (TAMs) and restrained cytotoxicity of CD8+ cytotoxic T lymphocytes (CTLs). The infiltrated MARCO+ TAMs can obstruct the development of CD8+ CTLs by impairing MHC‐I‐mediated neoantigen cross‐presentation. Mechanistically, MARCO up‐regulates the expression of SOCS1, which obstructs the kinase activity of JAK1, thereby downregulating MHC‐I expression through the inhibition of the JAK1‐STAT1‐NLRC5 signaling cascade. Further, MARCO blockade significantly facilitates ICB therapy in in vivo models by recovering tumor recognition and priming anti‐tumor CD8+ T cell responses. Taken together, these findings highlight MARCO as a highly desirable target in ICB‐refractory individuals for immunorecognition reignition and immunotherapy modulation.

## Graphical abstract

MARCO+ TAMs dominate restrained cytotoxicity of CD8+ CTLs and ICB resistance in RCC. Mechanically, MARCO impairs MHC‐I‐mediated neoantigen cross‐presentation via the SOCS1‐JAK1‐STAT1‐NLRC5 signaling cascade in TAMs. Targeted MARCO treatment boosts macrophage antigen cross‐presentation and empowers CD8+ T cells, thereby potentiating ICB efficacy.

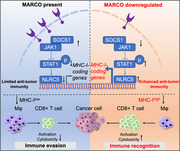

## 1 Introduction

Renal cell carcinoma (RCC) is the most common subtype of renal cancer, witnessing a steady surge in its global incidence.^[^ ^1^, ^2^ ^]^ RCC is also considered the most immune and vascularly infiltrated cancer type.^[^ ^3^, ^4^ ^]^ Immunotherapy‐based combinations in RCC have shown significant survival benefits, with data demonstrated by trials like RENOTORCH,^[^ ^5^ ^]^ CheckMate 9ER,^[^ ^6^ ^]^ and CheckMate 214.^[^ ^7^ ^]^ The introduction of immune checkpoint blockade (ICB) has heralded a notable breakthrough in the treatment of RCC, with ICB therapies now becoming key components of the therapeutic armamentarium in the frontline treatment scenarios. As ICB‐based therapies have changed the management of RCC over the years, investigators are now confronting the critical challenge of determining appropriate strategies when a patient's tumor gets resistance to immunotherapy. Tumors adopt a range of immunoevasive properties to evade immune recognition and elimination, which enable overt immune escape and resistance to ICB.^[^ ^8^ ^]^ Identifying and mitigating immunoevasive mechanisms may be devoted to devising clinically effective strategies for reigniting or reengaging the immune system in that ICB‐refractory tumor microenvironment (TME).

The basis for ICB failure involves multiple mechanisms, including lack of tumor neoantigens,^[^ ^9^, ^10^ ^]^ failure to reverse T cell exhaustion,^[^ ^11^, ^12^ ^]^ and intra‐tumoral presence of immunosuppressive immune cells, including tumor‐associated macrophages (TAMs).^[^ ^13^ ^]^ Immune system sentinels known as TAMs are thought to support ICB resistance by undermining anti‐tumor immunity and fostering tumor development.^[^ ^14^ ^]^ TAMs can directly^[^ ^15^, ^16^ ^]^ and indirectly suppress CD8+ tumor‐infiltrating CD8+ cytotoxic T lymphocytes (CTLs) function, drive immunosuppression through secretion of factors like interleukin‐10 (IL‐10),^[^ ^17^ ^]^ and promote tumor cell proliferation and extravasation by supporting vascularization and development of extracellular matrices.^[^ ^18^ ^]^ By creating a physical barrier that prevents immune effector cells from being recruited to the TME, TAMs can actively encourage immunological exclusion.^[^ ^15^, ^19^ ^]^ TAMs can impede the extravasation of effector T cells into the tumor, hinder their expansion, and reduce the viability of CD8+ CTLs.^[^ ^20^ ^]^ Furthermore, for CD8+ CTLs to be primed and activated, antigens must be presented by professional antigen‐presenting cells in complex with major histocompatibility complex (MHC) class I molecules. Thus, antigen processing and presentation defects by TAMs, may also contribute to driving T cell exhaustion and consequent resistance to ICB.^[^ ^21^, ^22^ ^]^ Clinically, the high density of TAMs in the TME of RCC has been reported to be associated with poor prognosis, signifying their role as central mediators of TME‐induced immunological suppression.^[^ ^23^, ^24^, ^25^ ^]^ The phenotypic diversity of both TAMs and T cells might be related to response and resistance to ICB in RCC. While targeting TAMs holds therapeutic potential, it crucially hinges on a more profound comprehension of the specific markers that is vital for TAM function. Thus, comprehensive analyses on RCC should be performed to elucidate the immune ignorance or immune exclusion mechanism of ICB resistance.

To better profile the immune infiltrations and understand mechanisms driving ICB resistance in advanced RCC patients, we analyzed RCC samples using single‐cell RNA sequencing (scRNA‐seq) pre‐ and post‐ICB therapy. We observed high infiltration of TAMs associated with limited expansion and effector differentiation of CD8+ CTLs in ICB‐resistant patients. We identify a specific TAM subset, MARCO+ TAMs, corresponding to a highly immunosuppressed TME. Functional studies demonstrate that MARCO+ TAMs were the key antigenic exposure sources that governed immune recognition and restrained the proliferation and cytotoxicity of CD8+ T cells. Mechanistically, MARCO+ TAM‐mediated immune evasion depends on the activation of suppressor of cytokine signaling 1 (SOCS1), which significantly downregulates MHC‐I expression via inhibition of the JAK1/STAT1/NLRC5 signaling cascade. In addition, MARCO blockade enhanced ICB responses and deterred tumor development in an orthotopic RCC preclinical model. Our data highlight the role of MARCO+ TAM in promoting immunological escape and uncover MARCO in TAM as a therapeutic target for advancing ICB responses in RCC. Currently, the unmet clinical need in advanced RCC lies in the development of therapeutic strategies for patients who fail immunotherapy combinations. This study provides a theoretical foundation for the development of innovative immunotherapeutic strategies for advanced RCC.

## 2 Results

### 2.1 Single‐Cell Atlas Illustrated the ICB Response‐Related TME Alterations in RCC Patients

**Figure 1:**
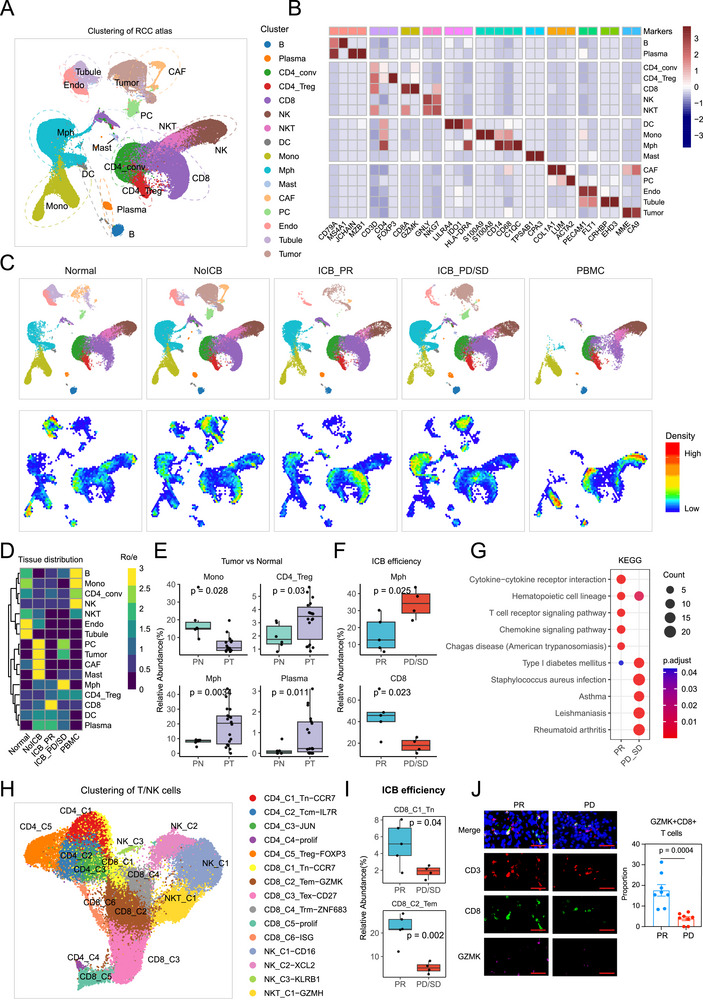
The dynamic single‐cell landscape of renal cell cancer (RCC) patients receiving immunotherapy. A) Uniform Manifold Approximation and Projection (UMAP) embedding of scRNA‐seq data for all 103345 cells. Five tissue types were included: adjacent normal (*n* = 6), immune checkpoint blockade (ICB) treatment‐naïve tumor (non‐ICB, *n* = 9), ICB sensitive tumor (partial response [PR], *n* = 5), ICB‐resistant tumor (progressive or stable diseases [PD/SD], *n* = 4) and peripheral blood mononuclear cell (PBMC) (*n* = 2) samples. B) Heatmap showing the RNA expression of marker genes used to define the 15 major cell types. C) UMAP embedding and cell abundance of scRNA‐seq data for normal, non‐ICB, ICB PR, ICB PD/SD and PBMC tissues. D) Heatmap displaying the distribution of 15 cell types across different tissue types (normal, non‐ICB, ICB PR, ICB PD/SD, and PBMC), as estimated by Ro/e. E) Boxplot comparing the abundances of monocytes, CD4+ regulatory T (Treg) cells, macrophages and plasma cells across adjacent normal (*n* = 9) and RCC tumor tissues (*n* = 18). F) Boxplot comparing the abundances of macrophages and CD8+ T cells across ICB sensitive (*n* = 5) and resistant tumor tissues (*n* = 4). The data are presented as a box‐and‐whisker graph (bounds of box: first to third quartile, bottom and top line: minimum to maximum, central line: median) for (E) and (F). G) Dot plot showing the results of Kyoto Encyclopedia of Genes and Genomes (KEGG) analyses of the differentially expressed genes in the ICB sensitive and resistant tumor tissues. H) UMAP embedding of scRNA‐seq data for 51301 T and natural killer (T/NK) cells. I) Boxplot comparing the abundances of CD8+ T cell subclusters across ICB sensitive (*n* = 5) and resistant tumor tissues (*n* = 4). The data are presented as a box‐and‐whisker graph (bounds of box: first to third quartile, bottom and top line: minimum to maximum, central line: median). J) Multiplex immunofluorescence of DAPI, CD3, CD8, and GZMK in ICB sensitive (*n* = 8) and resistant (*n* = 8) tumor tissues. Left: representative images; Right: statistical diagram. Scale bar, 50 µm. Data are presented as mean ± SEM. The statistical significance was tested via unpaired two‐sided Student's t test for (E), (F), (I), and (J).

In this study, to understand the cellular and molecular dominants of ICB response in RCC, we collected scRNA‐seq data of 18 RCC patients with or without ICB treatments (Table S1, Supporting Information). Most of the patients (*n* = 17) had a histology of clear cell renal cell carcinoma, and only one patient was diagnosed with papillary renal cell carcinoma. We obtained the single‐cell transcriptomes from 6 adjacent normal samples, 9 ICB treatment‐naïve tumor samples, 5 ICB‐sensitive tumor (partial response [PR]) samples, 4 ICB‐resistant tumor (progressive disease or stable disease [PD/SD]) samples and 2 peripheral blood mononuclear cell (PBMC) samples. These samples contributed a total of 103345 high‐quality single‐cell transcriptomes. Lineage‐specific and cluster‐enriched genes were rigorously cross‐checked against existing datasets using unsupervised clustering and manual annotation. Through this integrated approach, we obtained cell‐specific transcriptomic signatures that delineated 15 distinct clusters, encompassing diverse immune subsets including tumor cells, myeloid cells (dendritic cells [DC], monocytes, macrophages, and mast cells), stromal cells (cancer‐associated fibroblasts [CAFs], pericytes [PCs], tubular cells and endothelial cells [ECs]), lymphocytes (B/plasma cells, T cells, natural killer [NK] cells, and NK‐like T cells [NKT]), and lymphocytes (**Figure** **1**A,B; and Table S2, Supporting Information). All cell clusters were shared across samples, datasets and subgroups, indicating that there were no batch effects (Figure S1A,B, Supporting Information), although considerable patient‐specific heterogeneity was observed (Figure S1C, Supporting Information).

We then performed tissue distribution analysis to dissect the compositional changes in the ICB‐treated tumors and other tissue types, and observed a significant tissue‐specific predominant cell infiltration, characterized by EC and tubular cell infiltration in normal kidney tissue, the infiltration of stromal (PCs and CAFs) and tumor components in ICB‐naïve samples, the infiltration of macrophages in ICB‐resistant samples, CD8+ T cell infiltration in ICB‐sensitive samples and B cell, monocyte and NK cell infiltration in PBMC (Figure 1C,D).

Macrophage expansion was one of the most noticeable alterations seen in tumors, emphasizing its important role in modulating the RCC TME (Figure 1E). TAMs appear to be the dominant immune cell type infiltrated in tumor ecosystems and are generally polarized into M2‐type macrophages, which always function as triggers of tumor initiation and progression.^[^ ^26^ ^]^ Subsequent analyses further enhanced the relevance of the infiltration of macrophages and the diminishment of CD8+ T cells with immunotherapy resistance (Figure 1F). The relationship between several immune response‐related pathways and ICB response was also validated by Kyoto Encyclopedia of Genes and Genomes (KEGG) analysis. These pathways included the cytokine‐cytokine receptor interaction, the T cell receptor signaling pathway, and the chemokine signaling pathway, all of which were downregulated in ICB‐resistant samples. (Figure 1G). Taken together, the ICB‐resistant tumors process an immunosuppressive TME, featured by abnormally infiltrated macrophages as well as diminished CD8+ T cells, exhibiting enhanced immune escape potential.

Considering the robust correlation of T/NK lymphocyte functions with ICB response^[^ ^27^ ^]^ (Figure 1F), we deciphered the landscape in T/NK cells in the single‐cell RCC atlas, which led to an identification of 14 transcriptional states (Figure 1H; Figure S2A and Table S2, Supporting Information). In detail, CD4+ T cells were reclustered into 5 transcriptional states, including naïve (Tn, C1), central memory (Tcm, C2), and regulatory CD4+ T cells (Treg, C5); CD8+ T cells were further sub‐divided into naïve (Tn, C1), effector memory (Tem, C2), exhausted (Tex, C3), tissue‐resident memory (Trm, C4), proliferative (C4) and interferon‐stimulated gene‐related (C5) T cells (Figure 1H; Figure S2A, Supporting Information). We also identified three clusters of NK cells (defined by *CD16*, *CD56*, and *KLRB1*) and an NKT cell cluster (Figure 1H; Figure S2A and Table S2, Supporting Information). Tissue distribution analysis revealed a significantly preferential CCR7+CD8+ Tn and GZMK+CD8+ Tem cell infiltration in ICB‐sensitive tumors (Figure 1I; Figure S2B–E, Supporting Information), which have been well established as a major combater against tumors.^[^ ^28^ ^]^ In contrast, ZNF683+CD8+ Trm cells, KLRB1+ NK cells, and FOXP3+CD4+ Treg cells showed an infiltrating trend in ICB‐resistant tumors (Figure S2B–E, Supporting Information). ZNF683+CD8+ Trm cells represent a distinct subset of tumor‐specific memory T cells, for which contradictory functions have been established with high potency of both cytotoxic activation and exhaustion.^[^ ^29^ ^]^ Further analyses reinforced the correlation of T cell functions (activation, cytotoxicity, etc.) with ICB responses (Figure S2F,G, Supporting Information). We further performed multiplex immunofluorescence (mIF) of CD3, CD8 and GZMK in RCC tissues, which validated the preference of GZMK+CD8+ Tem cells in the ICB‐sensitive tumors (*p* < 0.001) (Figure 1J), supporting its essential role in protecting against immune escape of RCC. The reduction of tumor‐killing GZMK+CD8+ Tem cells and the accumulation of tumor‐resident ZNF683+CD8+ Trm cells indicate substantial remodelling of CD8+ T cell populations in ICB‐resistant RCC, of which the driving factors require in‐depth investigation.

We also investigated the relationships with tumor cells and stromal cells (Figures S3 and S4, Supporting Information). Subsequent clustering of tumor cells has pinpointed TMEM176B+ tumor cells as an indicator of ICB resistance (Figure S3A–C and Table S2, Supporting Information). Through the release of inflammasome activation, the cation channel TMEM176B has surfaced as a possible novel immunoregulatory target that might interfere with CD8+ T cell‐mediated tumor growth suppression.^[^ ^30^ ^]^ Prior research mostly concentrated on TMEM176B's roles in TAMs,^[^ ^31^ ^]^ while our studies further demonstrated the potential role of tumor‐derived TMEM176B in ICB resistance (Figure S3A–C, Supporting Information). The residual tumor cells after therapy were often regarded as therapy‐resistant cells, thus we compared the post‐ICB residual tumor cells with the pre‐ICB tumor cells to investigate the pathway related with ICB resistance (Figure S3D–F, Supporting Information). The results revealed that glycolysis/gluconeogenesis and amino acid metabolism pathways were significantly upregulated in post‐ICB residual tumor cells (Figure S3D–F, Supporting Information), emphasizing the contributions of hypoxia and metabolic reprogramming in ICB resistance of RCC.^[^ ^32^ ^]^ Clustering of stromal cells unravels the infiltration of FLT1+ ECs in ICB‐resistant tumors (Figure S4A–C, Supporting Information), which functioned as angiogenic ECs (Figure S4D, Supporting Information), and might be related with ICB resistance.

Together, we have constructed a comprehensive ICB response‐governed TME atlas of RCC, which illustrates an aggressive and immunosuppressive ecosystem with extensive cellular and molecular alterations in ICB‐resistant patients, including aberrant infiltration of macrophages, diminution of cytotoxic CD8+ T cells, and hypoxic and metabolic reprogramming of tumor cells.

### 2.2 The Transcriptional Diversity and Single‐Cell Trajectory of Myeloid Cells in RCC

**Figure 2:**
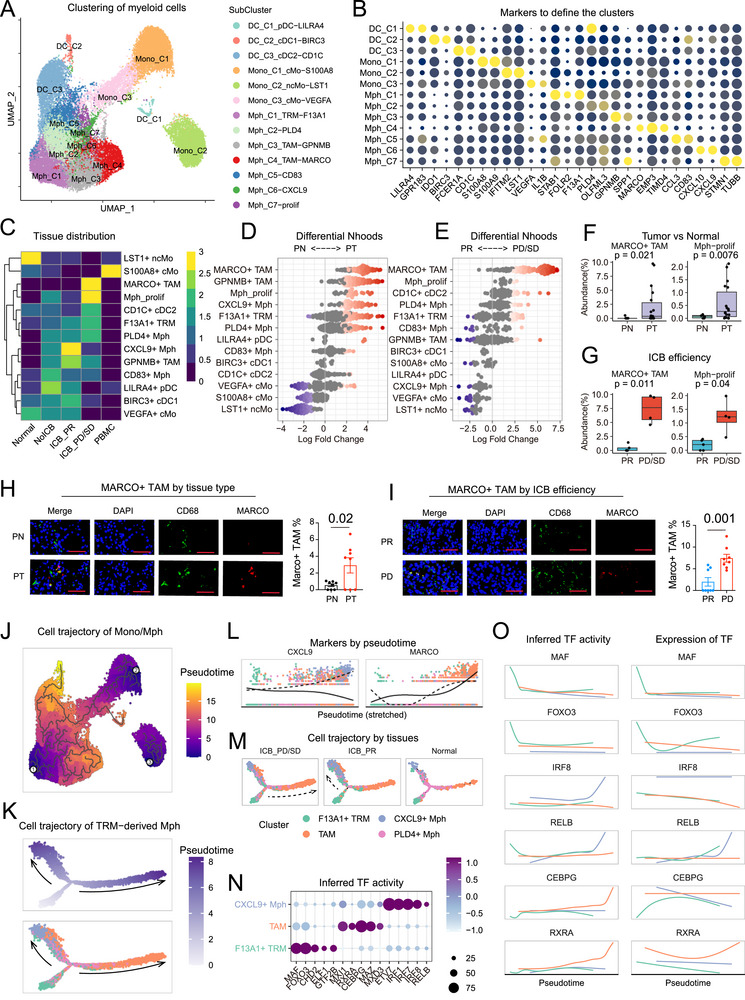
Landscape and cell trajectory of myeloid cells. A) Uniform Manifold Approximation and Projection (UMAP) embedding of scRNA‐seq data for 28340 myeloid cells. B) Dotplot showing the RNA expression of marker genes used to define the myeloid cell subclusters. C) Heatmap displaying the distribution of myeloid cell subclusters across different tissue types, as estimated by Ro/e. Five tissue types were included: adjacent normal, immune checkpoint blockade (ICB) treatment‐naïve tumor (non‐ICB), ICB sensitive tumor (partial response [PR]), ICB‐resistant tumor (progressive or stable diseases [PD/SD]) and peripheral blood mononuclear cell (PBMC) samples. D) Beeswarm plot showing the distribution of adjusted log2FC fold change (FC) in abundance between the tumor (*n* = 18) and normal (*n* = 9) tissues in Nhoods defined by Milo algorithm according to myeloid cell subclusters. E) Beeswarm plot showing the distribution of adjusted log2FC in abundance between the ICB sensitive (*n* = 5) and resistant (*n* = 4) tumors in Nhoods defined according to myeloid cell subclusters. F) Boxplot comparing the abundances of MARCO+ tumor‐associated macrophages (TAMs) and proliferating macrophages across adjacent normal (*n* = 9) and tumor (*n* = 18) tissues. G) Boxplot comparing the abundances of MARCO+ TAMs and proliferating macrophages across ICB sensitive (*n* = 5) and resistant (*n* = 4) tumor tissues. The data are presented as a box‐and‐whisker graph (bounds of box: first to third quartile, bottom and top line: minimum to maximum, central line: median) for (F) and (G). H) Representative images and statistical diagram of multiplex immunofluorescence staining of DAPI, CD68 and MARCO in adjacent normal (*n* = 8) and tumor (*n* = 8) tissues. Scale bar, 50 µm. Data are presented as mean ± SEM. I) Representative images and statistical diagram of multiplex immunofluorescence staining of DAPI, CD68 and MARCO in ICB sensitive (*n* = 8) and resistant (*n* = 8) tumors. Scale bar, 50 µm. Data are presented as mean ± SEM. J) Cell trajectories of monocytes/macrophages inferred by Monocle 3. K) Pseudotime and cell trajectories of tissue‐resident macrophages (TRM)‐derived macrophages inferred by Monocle2. L) Expression of *CXCL9* and *MARCO* by pseudotime in cell trajectories of TRM‐derived macrophages. M) Cell trajectories of TRM‐derived macrophages inferred by Monocle2 in the ICB‐sensitive, ICB‐resistant and normal groups. N) Differential transcription factor activity between F13A1+ TRM cells, TAMs and CXCL9+ macrophages. O) Dynamic changes in transcription factor activity (left) and expression (right) over time. The unpaired two‐sided Student's t test was used for (F), (G), (H), and (I).

Considering that myeloid cells significantly infiltrate the RCC ecosystem and demonstrate a significant relation with ICB resistance, we further re‐analyzed myeloid subpopulations by sub‐clustering the identified 28340 myeloid cells (**Figure** **2**A). Based on high expression of HLA‐DRs and low expression of CD14, we observed three clusters of DCs, including cDC1 (*IDO1/LAMP3*), cDC2 (*CD1C/FCER1A*), and pDC (*LILRA4/GPR183*) (Figure 2B; Figure S5A–C, Supporting Information). Given that macrophages are involved in angiogenic pathways that support tumor development^[^ ^33^, ^34^, ^35^, ^36^ ^]^ and are linked to tyrosine kinase inhibitor outcome,^[^ ^37^ ^]^ their phenotypic variability is of significant relevance in RCC. Macrophages were clearly delineated by *CD14*, *CD68*, *CD163*, and *MRC1*. The Mph_C1, which highly expresses the resident‐like markers *FOLR2*, *MRC1* and *F13A1* is designated as tissue‐resident macrophages (TRMs).^[^ ^38^ ^]^ TAMs (Mph_C3 and Mph_C4) were characterized by *TREM2*, *MARCO* and *SPP1* (Figure 2B; Figure S5A–C and Table S2, Supporting Information).

Notably, we found that macrophages were preferentially enriched in tumor tissues, while LST1+ non‐classical monocytes were predominantly present in the adjacent normal tissue (Figure 2C–F). Among all clusters, MARCO+ TAMs were discovered to be strikingly abundant in tumors relative to adjacent normal tissues (PT versus PN, *p* = 0.02) (Figure 2C‐D,F), which was consistent with previous studies.^[^ ^39^ ^]^ In macrophages, the class A scavenger receptor MARCO is well‐characterized and valued for its function in identifying and eliminating pathogens by recognizing pathogen‐associated molecular patterns.^[^ ^40^, ^41^ ^]^ MARCO is an important prototypic marker of TAMs, as evidenced by recent research showing that increased MARCO expression is linked to a worse prognosis of a variety of malignancies.^[^ ^42^, ^43^, ^44^, ^45^, ^46^ ^]^

We next investigated the dynamic statuses of these various TAMs according to ICB exposure in order to gain a better understanding of the roles they play in ICB response (Figure 2E–G). MARCO+ TAMs were enriched in the ICB non‐responders compared with those responders (PD versus PR: *p* = 0.01), and also were upregulated post ICB treatments compared with treatment‐naive samples in both internal and external datasets^[^ ^47^ ^]^ (Figure S5D,E, Supporting Information). Additionally, the phenotype of TAMs in ICB non‐responders was more proliferative (Figure 2E–G). Furthermore, mIF also confirmed the accumulation of MARCO+ TAMs in the ICB non‐responder group (Figure 2H,I). The expression of immunosuppressive markers confirmed the definite TAM state of this special cell cluster (Figure S5F, Supporting Information). Collectively, these observations pointed to the extensive reprogramming of TAMs in RCC TME. Exactly, failed ICB therapy elicited higher surface MARCO on TAMs, which contributes to a trending increase of MARCO+ TAMs, highlighting the potential role of MARCO and MARCO+ TAMs in immune inhibition and tumor growth promotion. Since MARCO has a detrimental immunomodulatory effect on various malignancies, we decided to concentrate on these MARCO+ TAMs and how they affect ICB resistance in RCC.

We used Monocle3 to create single‐cell trajectories of monocytes/macrophages in order to investigate the source of the abnormally infiltrating TAMs in RCC. Through the Monocle3 algorithm, we uncovered three distinct developmental trajectories: 1) Monocyte‐derived macrophages were represented by the first trajectory (Branch 1), where S100A8+ classical monocytes predominated earlier while VEGFA+ classical monocytes emerged later; 2) TRM‐derived TAMs were represented by the second trajectory (Branch 2), which mapped F13A1+ TRMs at the initial timepoint and TAMs and CXCL9+ macrophages at later developmental timepoints; 3) the third trajectory (Branch 3) corresponded to LST1+ non‐classical monocytes (Figure 2J). Our findings were in line with earlier data, suggesting that the majority of TAMs in the RCC population may be mostly produced from TRM.^[^ ^38^ ^]^ To further understand the developmental trajectories of TRM‐derived macrophages, we conducted additional investigations (Figure 2K−M). While CXCL9+ macrophages and TAMs divided into two distinct cell fates, the majority of F13A1+ TRMs held a prebranch state, according to our pseudotime analysis, confirming F13A1+ TRMs as a significant alternative source of macrophages (Figure 2K−M).

We examined the dynamic transcription factors (TFs) fluctuations throughout the establishment of various lineages in order to discover the reasons that drive these developmental shifts (Figure 2N,O). We found a series of TF reprogramming during the TRM developments, with both the expression levels and TF activity of SPIB, IRF8 and RELB enhanced in CXCL9+ macrophages, as well as the expression levels and TF activity of RXRA and CEBPG upregulated in the trajectory into TAMs (Figure 2N,O). RXRs have been reported to control macrophage expansion and contribute to ovarian cancer progression.^[^ ^48^ ^]^ Multiple members of CCAAT/enhancer‐binding protein (CEBP) family, including CEBPA and CEBPD, have been implicated with potential in modulating macrophage activation,^[^ ^49^, ^50^ ^]^ whereas we reported CEBPG as a new modulator of TAM development.

Taken together, through in‐depth investigations on the transcriptional diversity and developmental trajectory of myeloid cells, we found a significant infiltration of MARCO+ TAMs driven by dynamic TF reprogramming in ICB‐resistant RCCs.

### 2.3 MARCO+ TAMs were Associated with Immunotherapy Resistance in RCC via Obstructing the Development of CD8+ T Cells

**Figure 3:**
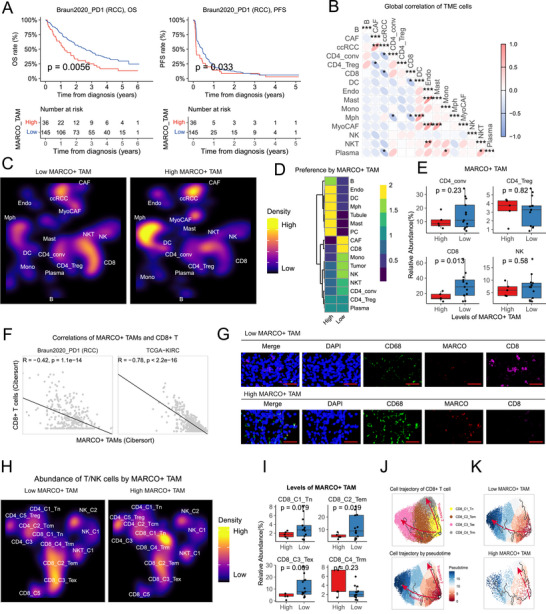
The Landscape of renal cell cancer (RCC) ecosystem altered by MARCO+ tumor‐asssociated macrophages (TAMs). A) Overall survival (OS) and progression‐free survival (PFS) in the immunotherapy cohort (Braun2020 PD1 cohort) stratified by the number of MARCO+ TAMs. The numbers of MARCO+ TAMs were inferred via the single sample gene set variation analysis (ssGSVA) method. B) Heatmap displaying the correlations across all cell types in the RCC single‐cell atlas. Significance of correlation: *, *p* < 0.05; **, *p* < 0.01; ***, *p* < 0.001. C) Uniform Manifold Approximation and Projection (UMAP) embedding and cell abundance of all cell lineages in RCC samples divided by the levels of MARCO+ TAMs. D) Heatmap displaying the distribution of all cell lineages in RCC samples with high (*n* = 5) and low (*n* = 13) levels of MARCO+ TAMs. E) Boxplot comparing the abundance of CD4+ T, CD4+ regulatory T (Treg), CD8+ T and natural killer (NK) cells in RCC samples with high (*n* = 5) and low (*n* = 13) levels of MARCO+ TAMs. The data are presented as a box‐and‐whisker graph (bounds of box: first to third quartile, bottom and top line: minimum to maximum, central line: median). F) Correlation of MARCO+ TAMs and CD8+ T cells in bulk RNA‐seq datasets. Two datasets were included: Braun2020 PD1 cohort and the The Cancer Genome Atlas (TCGA) kidney renal clear cell carcinoma (KIRC) cohort. The abundance of MARCO+ TAMs and CD8+ T cells were inferred via the CIBERSORTx method. G) Representative images of multiplex immunofluorescence comparing the abundance of CD8+ T cells in RCC samples with high and low levels of MARCO+ TAMs. Four markers were stained: DAPI, CD68, MARCO and CD8. H) UMAP embedding and cell abundance of CD8+ T cells in RCC samples divided by the levels of MARCO+ TAMs. Scale bar, 50 µm. I) Boxplot comparing the abundance of CD8+ T cell subclusters in RCC samples with high (*n* = 5) and low (*n* = 13) levels of MARCO+ TAMs. The data are presented as a box‐and‐whisker graph (bounds of box: first to third quartile, bottom and top line: minimum to maximum, central line: median). J,K) Monocle3 inferred cell trajectories of CD8+ T cells (J) in all tissues and (K) in RCC samples with high and low levels of MARCO+ TAMs. The Log‐rank test was used for (A). The unpaired two‐sided Student's t test was used for (E) and (I). Person's correlation analysis was used for (B) and (F).

In accordance with the infiltrating preference of MARCO+ TAMs in ICB non‐responders, the bulk RNA‐seq cohort also indicated a correlation between greater levels of MARCO+ TAMs and significantly unfavorable prognosis in RCC patients with ICB treatments (overall survival: *p* = 0.006; progression‐free survival: *p* = 0.03) (**Figure** **3**A; and Table S3, Supporting Information). These data proposed a robust correlation between MARCO+ TAMs and ICB resistance; thus, we sought to investigate the ICB‐resistant mechanisms mediated by MARCO+ TAMs.

We initially looked at the correlations across all cell lineages to determine the phenotypic interactions of MARCO+ TAMs with the different components in the TME of RCC. We found that MARCO+ TAMs and CD8+ T cells had a negative association (R = ‐0.51, *p* = 0.029) (Figure 3B). We categorized the tumors according to the infiltration of MARCO+ TAMs and compared the compositional changes in the TME components between the MARCO+ TAM‐high and ‐low groups (Figure 3C). The results elucidated that one of the most dramatically changed TME components was CD8+ T cells, which were drastically decreased (*p* = 0.01, Figure 3C–E). Given that the global cell interaction analyses pinpointed the interference of MARCO+ TAMs with CD8+ T cells, we then looked into how MARCO+ TAMs affected CD8+ T cells. We first estimated the correlation of CIBERSORTx‐inferred abundance of MARCO+ TAMs and CD8+ T cells in bulk RNA‐seq data (Table S3, Supporting Information), which validated the negative association between MARCO+ TAMs and CD8+ T cells in both ICB‐treated and the Cancer Genome Atlas (TCGA) kidney renal clear cell carcinoma (KIRC) cohorts (Figure 3F). We further adopted mIF tests to confirm the connections between MARCO+ TAMs and CD8+ T cells, which also illustrated a lower number of CD8+ T cells in MARCO+ TAM‐high tumors (Figure 3G). These results were consistent with the previous studies, among which MARCO+ TAMs have been reported to facilitate immune escape, via the promotion of regulatory T cells and inhibition of CD8+ T cells.^[^ ^51^ ^]^ Notably, significantly fewer CD8+ T subpopulations, including CD8+ Tem (*p* = 0.019), CD8+ Tex (*p* = 0.0085) and proliferative CD8+ T cells (*p* = 0.04) were detected in the MARCO+ TAM‐high group (Figure 3H,I). In contrast, the resting CD8+ Trm cells showed a trend toward increased infiltration in the MARCO+ TAM‐high group (Figure 3H,I). This contributed to a conflicting phenomenon in the CD8+ T cell clusters, indicating that CD8+ T cell cytotoxic and expanding capacity might be hindered by MARCO+ TAMs.

We further adopted Monocle3 to examine the CD8+ T developmental trajectory and further dissect the impacts of MARCO+ TAMs on it (Figure 3J,K; Figure S6, Supporting Information). The Monocle3 analyses illustrated two distinct developmental trajectories: one starts at CD8+ Tn cells, is subsequently activated into CD8+ Tem cells, and finally ends at CD8+ Tex cells; and another trajectory starts at CD8+ Tn cells and ends at resting CD8+ Trm cells (Figure 3J). When classified by the levels of MARCO+ TAMs, the developmental trajectory of CD8+ T cells exhibited distinct patterns: 1) in the MARCO+ TAM‐low group, the CD8+ T cells could develop normally, with abundant cells at the activated CD8+ Tem state; 2) in the MARCO+ TAM‐high group, numerous CD8+ T cells have been rested at the Trm state, while the cells at activated effector state reduce significantly (Figure 3J,K; Figure S6A,B, Supporting Information). These findings indicated that the developmental process of CD8+ T cells might be impeded by MARCO+ TAMs, which significantly restrain the activation and expansion of CD8+ T cells, therefore contributing to the conflicting phenomenon of accumulated resting Trm cells coupled with lacked activating Tem cells.

Together, we found that MARCO+ TAMs might be related with restrained activation of CD8+ T cells, resulting in impaired CD8+ T cell‐mediated cytotoxicity as well as ICB resistance. Further developmental trajectory analyses revealed that the mechanisms of immune escape mediated by MARCO+ TAMs might be achieved by disrupting the development of CD8+ Tn cells into effector T cells.

### 2.4 RCC‐Conditioned MARCO+ TAMs Suppress CD8+T Cell Activation and Anti‐Tumor Immunity

**Figure 4:**
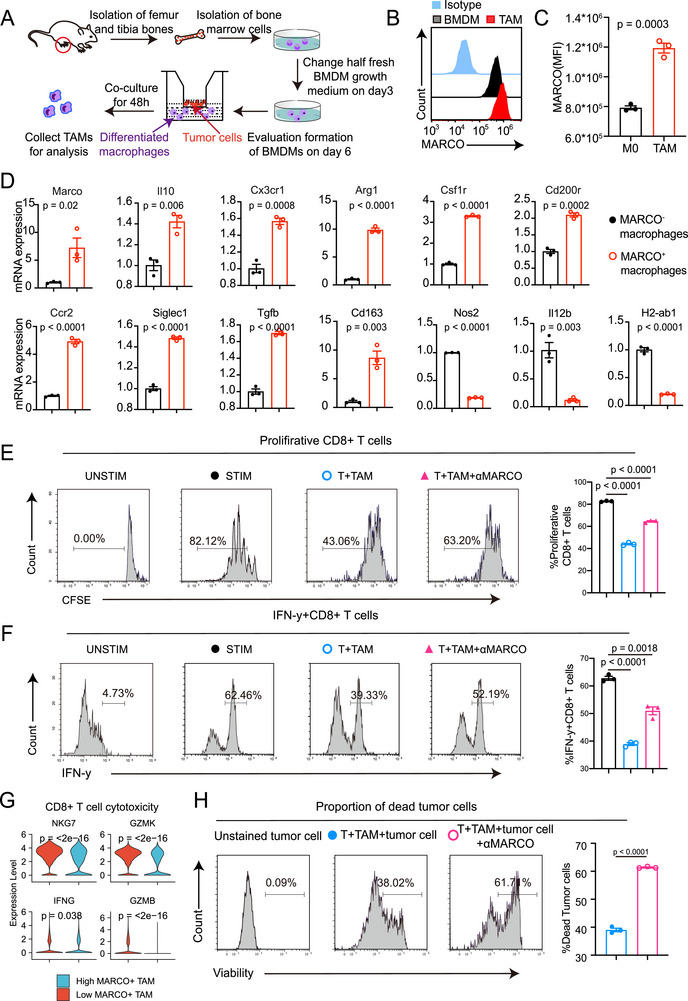
Tumor‐conditioned MARCO+ tumor‐asssociated macrophages (TAMs) have an immune‐suppressive phenotype, and targeting results in the reactivation of CD8+ T‐cell functionality. A) Methodological scheme for generating TAMs. Purified monocytes from mice were differentiated in MCSF and later conditioned with renal cancer cells and assessed for their MARCO expression. B,C) Representative histograms and mean fluorescence intensity (MFI) were shown and statistical analyses were performed (*n* = 3). Data are presented as mean ± SEM. D) Phenotyping of MARCO+ macrophages compared with MARCO‐ macrophages by qPCR (*n* = 3). Data are presented as mean ± SEM. E,F) CD8+ T cells were cocultured with TAMs incubated with anti‐MARCO Ab and assessed for proliferation and IFN‐y production (*n* = 3). Data are presented as mean ± SEM. G) Expression of cytotoxic markers (such as *NKG7*, *GZMK*, *GZMB*, and *IFNG*) in CD8+ CTLs according to infiltration of MARCO+ TAMs in the RCC single‐cell dataset. H) CD8+ T cells were cocultured with TAMs incubated with anti‐MARCO Ab and their tumor cell killing capacity was measured. CFSE labeled renal cancer cells were added to the T cells and percentage of dead tumor cells was measured by flow cytometry (*n* = 3). Data are presented as mean ± SEM. The unpaired two‐sided Student's t test was used for (C), (D), (E), (F), (H). Two‐sided Wilcoxon test was used for (G).

We next investigated the mechanistic interactions between the MARCO+ TAMs and cytotoxic lymphocytes in vitro. Mouse bone‐marrow‐derived macrophages (BMDMs) were cocultured with renal cancer cell line Renca for 48 h to be conditioned into TAMs, during which tumor cells and BMDMs were cultured separately using transwell inserts to prevent direct contact. Through this approach, we verified the upregulated MARCO expression on TAMs compared to M0 BMDMs (**Figure** **4**A–C). A subclass of immunosuppressive TAMs has been shown to be represented by MARCO, which is linked to other pro‐tumoral markers such IL4R, CHIA, CD163, and EMT markers.^[^ ^52^ ^]^ Quantitative reverse transcription polymerase chain reaction (RT‐qPCR) of these RCC‐derived TAMs showed the similar pattern, with MARCO expression linked to M2‐like phenotypic genes (*Ccr2*, *Cx3cr1*, *Tgfb* etc.) and M2‐connected genes (*Il10*, *Csf1r*, *Arg1*, etc.), but not M1 genes (*Nos2*, *Il12b*, *H2‐ab1*, etc.) (Figure 4D).

Given the immunosuppressive profile of MARCO+ TAMs and the association between MARCO and T cell exhaustion and regulatory T cells,^[^ ^45^ ^]^ we next assessed the MARCO‐mediated interaction between TAMs and CD8+ CTLs. Purified CD8+ CTLs isolated from mice spleens were cocultured with RCC‐conditioned TAMs. We found that when cocultured with RCC‐derived TAMs, CD8+ CTLs exhibited a suppressed phenotype with restrained proliferation and IFN‐γ production. After treating TAMs with anti‐MARCO antibody, CD8+ CTLs acquired enhanced proliferation activity and secreted strikingly higher levels of IFN‐γ than those without treatment (Figure 4E,F), suggesting that MARCO blockade recovers the inhibited CD8+ CTL activity. To evaluate the tumor‐killing capacity of these CD8+ CTLs following coculture with TAMs, we introduced the CD8+ CTLs into Renca cell cultures and subsequently conducted cytotoxicity assays. In the single‐cell dataset, infiltration of MARCO+ TAMs was also related with impaired cytotoxicity of CD8+ CTLs, with downregulated cytotoxic markers, such as *NKG7*, *GZMK*, *GZMB*, and *IFNG* (Figure 4G). Besides, we also transferred the single‐cell annotation in a RCC spatial transcriptome dataset^[^ ^53^ ^]^ and investigated the in situ interaction pattern of MARCO+ TAMs and CD8+ T cells. The results revealed that there was an excluding interaction pattern between MARCO+ TAMs and CD8+ T cells (Figure S7A, Supporting Information), which could also be validated by the in situ mIF assays (Figure S7B, Supporting Information). In line with the phenotypic findings, our observations revealed that the TAMs pretreated with MARCO antibody are capable of activating CD8+ CTLs and inducing substantial tumor cell apoptosis (Figure 4H). Collectively, these findings indicate that TAMs rendered immunosuppression by MARCO can impair CD8+ CTL activation and tumor‐killing capabilities. Conversely, targeting MARCO on TAMs can promote the reactivation of CD8+ CTLs, thereby enhancing the antitumor responses.

These data validated the immunosuppressive role of MARCO+ TAMs, which facilitate immune escape via significantly hindering the activation of the CD8+ T cells, highlighting the importance of MARCO‐targeted therapy in the restoring of anti‐tumor immunity.

### 2.5 MARCO Impedes Immune Recognition of RCC Tumor Cells by Impairing Antigen Presentation

**Figure 5:**
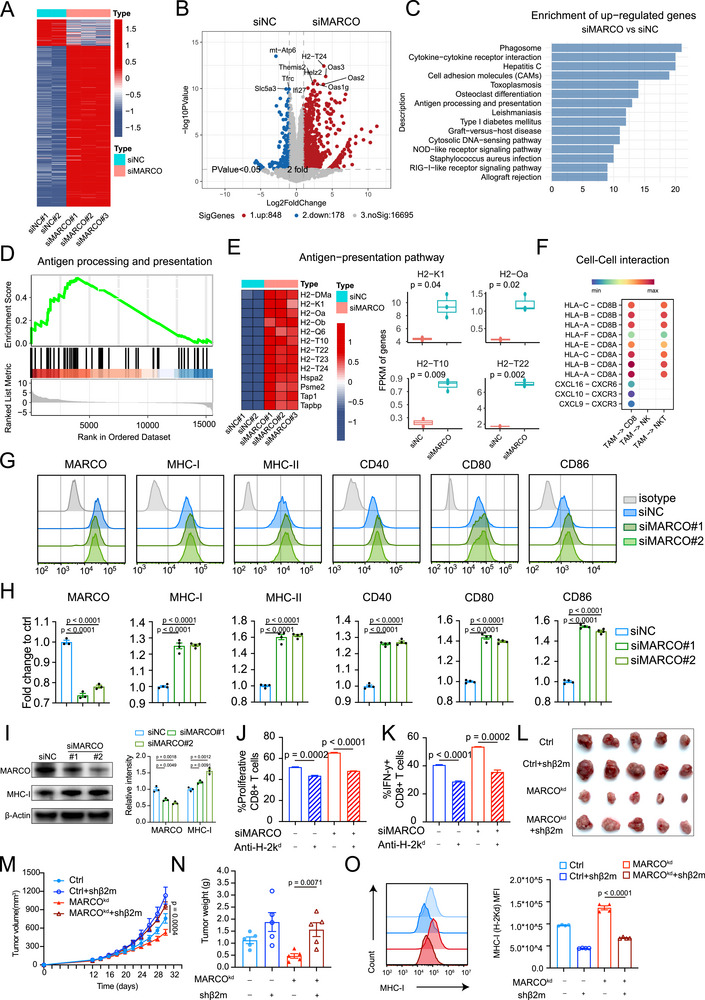
MARCO+ tumor‐asssociated macrophages (TAMs) restricted CD8+ T‐cell cytotoxicity partially due to impaired major histocompatibility complex (MHC) class I expression. A,B) Transcriptome sequencing was performed on TAMs from control group (siNC, *n* = 2) and knockdown group (siMARCO, *n* = 3). A) shows the differential gene heat map of sequencing results and B) shows the volcano map. C) Top 15 pathways changed in TAMs from siMARCO group compared with siNC group revealed by Kyoto Encyclopedia of Genes and Genomes (KEGG) pathway enrichment analysis. D) Gene‐set enrichment analysis (GSEA) results showed that MARCO might be closely related to antigen presentation pathway. E) Left: Heat map shows genes involved in antigen presentation pathway in TAMs from siMARCO group versus siNC group. Right: FPKM expression of H2‐K1, H2‐Oa, H2‐T10, and H2‐Ob quantified by RNA‐seq from siNC (*n* = 2) or siMARCO (*n* = 3) TAMs. The data are presented as a box‐and‐whisker graph (bounds of box: first to third quartile, bottom and top line: minimum to maximum, central line: median). F) The intercellular interaction analyses between TAMs and CD8+ T cells. G,H) Flow cytometric analysis of surface marker expression of MHC‐I, MHC‐II, CD40, CD80, and CD86 on TAMs from siNC and siMARCO group. MFI were shown and statistical analyses were performed (*n* = 4). Data are presented as mean ± SEM. I) Left: Western blot of MHC‐I in TAMs from siNC and siMARCO groups using β‐Actin as loading control. Right: The quantitation of band intensity (*n* = 3). Data are presented as mean ± SEM. J,K) TAMs from siNC and siMARCO group were co‐cultured with CFSE‐labeled CD8+ T cells from spleens of mice with control isotype or H‐2K^d^ blocking antibody. CD8+ T‐cell proliferation was measured by CFSE dilution as presented in (J), and the secretion of the cytokines IFN‐γ by CD8+ T cells was analyzed by flow cytometry as presented in (K). L–O) Renca cells mixed with TAMs (MARCO and/or MHC‐I knockdown) (2:1 ratio) were subcutaneously inoculated into BALB/c mice to establish tumor models. Tumor image (L) and volume (M), and weight (N) are shown. *n* = 5 per group. Data are presented as mean ± SEM. (O) MHC‐I expression on TAMs in different groups are determined by flow cytometry (*n* = 4). Data are presented as mean ± SEM. The unpaired two‐sided Student's t test was used for (E), (H), (I), (J), (K), (M), (N), and (O).

To investigate the molecular mechanism by which MARCO+ TAMs impact the CD8+ CTLs infiltration and function, small interfering RNAs were given to MARCO knockdown (siMARCO) on TAMs, which were then subjected to RNA sequencing (Table S4, Supporting Information). According to subsequent differentially expressed genes (DEGs) and enrichment studies, antigen processing and presentation, phagocytosis and inflammation were among the pathways in which TAMs from the siMARCO group were enriched (**Figure** **5**A–C). The gene‐set enrichment analyses (GSEA) also showed that the antigen processing and presentation pathway was significantly upregulated in MARCO‐knockdown group (Figure 5D). Antigen presentation‐related genes (e.g., *H2‐DMα*, *H2‐K1*, *H2‐Oa*, *H2‐Ob*, *H2‐T10*, *H2‐T22*, *H2‐T23*, *H2‐T24*, and *Tap1*) were also upregulated in TAMs from siMARCO group (Figure 5E). The intercellular interaction analyses suggested that TAMs activate CD8+ T cells via the MHC‐I:CD8 axis (Figure 5F). In accordance, flow cytometry analysis illustrated that TAMs from siMARCO group manifested advantages in the antigen‐presentation machinery, as evidenced by higher expression of antigen‐presenting molecules, including MHC‐I and MHC‐II in addition to surface costimulatory molecules, such as CD86, CD80, and CD40 (Figure 5G‐H). We further sorted MARCO+ and MARCO‐ TAMs from RCC tumor tissues and performed transcriptome analyses, which confirmed the downregulation of antigen‐presentation pathway and MHC‐I genes (*HLA‐A* and *HLA‐B*) in MARCO+ TAMs (Figure S8A–G, Supporting Information). The RCC single‐cell atlas also showed a dynamic downregulation of MHC‐I genes (*HLA‐A* and *HLA‐B*) in MARCO+ TAMs (Figure S8H–J, Supporting Information). After phagocytosing exogenous antigen, professional antigen‐presentation cells should load and cross‐present it in order to interact with CD8+ T cells via MHC‐I.^[^ ^54^ ^]^ One prevalent mechanism driving acquired resistance to immunotherapy is the downregulation of MHC‐I, reinforcing the critical role of antigen‐presentation immune camouflage. Western blot analysis also supports our findings that MARCO negatively regulates MHC‐I expression (Figure 5I). Therefore, we next focus on MHC‐I expression on MARCO+ TAMs to investigate their role in tumor antigen cross‐presentation.

Efficient immune recognition of tumor cells is the foundation of anti‐tumor immunity; thus, tumor self‐antigen presentation or cross‐presentation by immune cells such as macrophages are vital for immunity‐based therapies.^[^ ^55^ ^]^ However, the antigen‐presentation ability of TAMs is often impaired, which results in reduced T‐cell activation and proliferation.^[^ ^56^ ^]^ Drawing from these findings, we further assessed how MHC‐I presentation by MARCO+ TAMs contributes to T cell dysfunction. We established an in vitro coculture system, where TAMs (pretreated with MHC‐I blocking antibody and/or siMARCO) were co‐cultured with activated T cells. We noticed that MHC‐I blockade induced significantly lower proportion of proliferative and IFN‐γ+CD8+ CTLs with the presence of MARCO+ TAMs. Besides, MHC‐I blockade partially attenuated CD8+ CTLs proliferation and IFN‐γ expression in the setting of MARCO‐silenced TAMs (Figure 5J,K). Next, MARCO and/or MHC‐I on TAMs were blocked by shRNA in vivo. MHC‐I blockade considerably attenuated tumor growth inhibition brought on by MARCO knockdown in in vivo models (Figure 5L–N). Flow cytometry results confirmed the mean fluorescence intensity (MFI) of MHC‐I molecules on TAM surface was significantly downregulated by MHC‐I blockade in TAMs (Figure 5O). In the meantime, MHC‐I blocking could partly hinder the recovery of tumor‐infiltrating CD8+ CTLs (both IFN‐γ+CD8+ CTLs and GZMB+CD8+ CTLs) caused by MARCO‐knockdown TAMs (Figure S9, Supporting Information), which is consistent with the in vitro findings.

Therefore, these results suggest that MARCO+ TAMs could restrain the MHC‐I machinery and compromise the antigen cross‐presentation and tumor‐recognition capacity of TAMs, thereby impeding CD8+ CTL immunity in RCC.

### 2.6 MARCO Restrains Antigen Cross‐Presentation via SOCS1‐Driven Suppression of JAK1‐STAT1‐NLRC5 Signaling

**Figure 6:**
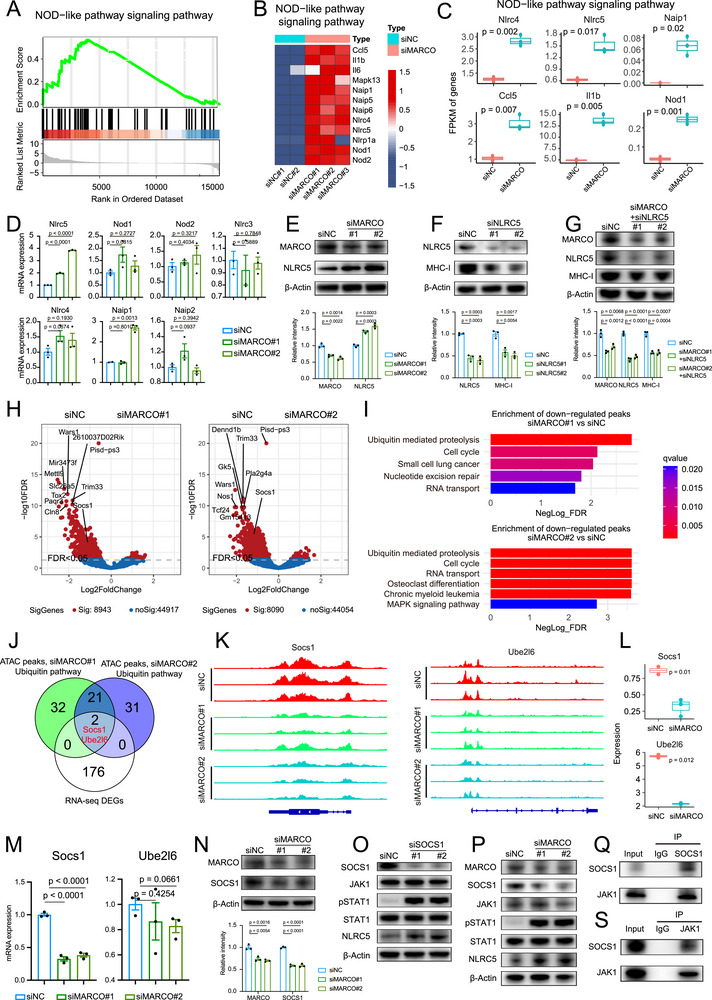
MARCO inhibits JAK1/STAT1/NLRC5 signaling by enhancing the activity of SOCS1. A) The Kyoto Encyclopedia of Genes and Genomes (KEGG) pathway enrichment analysis of the differential genes in the transcriptome revealed differences in Nucleotide Oligomerization Domain like (NOD‐like) receptor signaling pathway. B) Heatmap of genes involved in NOD‐like receptor pathway in MARCO knockdown or control TAMs. C) Fragments per kilobase of exon model per million (FPKM) expression of *IL1B*, *NOD2*, *NLRC4* and *NLRC5* quantified by RNA‐seq from siNC (*n* = 2) and siMARCO (*n* = 3) TAMs. The data are presented as a box‐and‐whisker graph (bounds of box: first to third quartile, bottom and top line: minimum to maximum, central line: median). D) Quantitative reverse‐transcription PCR (RT‐qPCR) was used to detect NOD‐like receptor mRNA levels in MARCO knockdown or control TAMs (*n* = 3 per group). Data are presented as mean ± SEM. E) Up: Western blot of NLRC5 in TAMs from siNC and siMARCO groups using β‐Actin as loading control. Down: The quantitation of band intensity (*n* = 3). Data are presented as mean ± SEM. F) Up: Western blot of MHC‐I in TAMs from siNC and siNLRC5 groups using β‐Actin as loading control. Down: The quantitation of band intensity (*n* = 3). Data are presented as mean ± SEM. G) Up: Western blot of MHC‐I in TAMs from siNC and siMARCO+siNLRC5 groups using β‐Actin as loading control. Down: The quantitation of band intensity (*n* = 3). Data are presented as mean ± SEM. H) ATAC‐seq volcano‐plot showing chromatin accessibility of DEGs detected in siNC and siMARCO TAMs. I) KEGG pathway enrichment for DEGs between siNC and siMARCO TAMs. J) Overlap of downregulated DEGs between the analysis of RNA‐seq and ATAC‐seq from siNC and siMARCO TAMs. K) Genome‐browser view of the ATAC‐seq peaks of Socs1 and Ube2l6 from siNC and siMARCO TAMs. L) Boxplot comparing the expression of Socs1 and Ube2l6 from siNC (*n* = 2) and siMARCO (*n* = 3) TAMs. The data are presented as a box‐and‐whisker graph (bounds of box: first to third quartile, bottom and top line: minimum to maximum, central line: median). M) RT‐qPCR analyses of *Socs1* and *Ube2l6* mRNA levels in MARCO knockdown or control TAMs (*n* = 3). Data are presented as mean ± SEM. N) Western blot of SOCS1 in TAMs from siNC and siMARCO groups using β‐Actin as loading control (*n* = 3). Data are presented as mean ± SEM. O) Western blot of JAK1, STAT1, p‐STAT1 and NLRC5 in TAMs from siNC and siSOCS1 groups using β‐Actin as loading control (*n* = 3). P) Western blot of SOCS1, JAK1, STAT1, p‐STAT1, and NLRC5 in TAMs from siNC and siMARCO groups using β‐Actin as loading control (*n* = 3). Q–S) The endogenous interaction between SOCS1 and JAK1 were detected by co‐immunoprecipitation assay (*n* = 3). The unpaired two‐sided Student's t test was used for (C), (D), (E), (F), (G), (L). (M), and (N).

Given that MARCO ablation in TAMs increases MHC‐I molecule expression and recovers cytotoxic T cell recruitment, we next interrogated the mechanisms how MHC‐I expression is inhibited. Subsequent analysis of the RNA‐seq data revealed that MARCO knockdown significantly activated the NOD‐like receptor signaling pathway in TAMs (**Figure** **6**A–C). The NOD‐like receptors (NLRs), primarily expressed in antigen‐presentation cells including macrophages and DCs, have been donated to recognize distinct endogenous or exogenous antigens and trigger inflammatory responses.^[^ ^57^ ^]^ Thus, the downregulation of NLR pathways in MARCO‐high group might indicate its involvement in the remodeling of the pro‐tumoral phenotype for TAMs. According to RT‐qPCR analysis of TAMs, the siMARCO group's mRNA levels of NLRs, including *Nlrc5*, *Nod1*, *Nod2*, *Nlrc4*, and *Naip2*, showed an increasing trend in comparison to the siNC group (Figure 6D). Among which, *Nlrc5* demonstrated the most significant upregulation (*p* < 0.0001).

NLRC5, a member of the NLR family, acts as a trans‐activator of the MHC‐I genes. It cooperates with transcription factors that connect with the conserved MHC‐I promoter regulatory elements, including the SXY module, to specifically associate with and trans‐activate MHC‐I promoters.^[^ ^58^, ^59^, ^60^, ^61^ ^]^ The Western blot analysis validated that the knockdown of MARCO in TAMs increased the protein levels of NLRC5 significantly (Figure 6E). Conversely, the NLRC5‐knockdown TAMs showed a significant reduction in the protein levels of MHC‐I molecules (Figure 6F). Furthermore, the knockdown of NLRC5 could further significantly impair the recovery of MHC‐I molecules caused by MARCO knockdown in TAMs (Figure 6G). These data suggested that MARCO can disrupt tumor immune recognition by blocking the NLRC5‐MHC‐I axis through NLRC5 downregulation, impairing antigen cross‐presentation in TAMs.

To further elucidate the mechanisms underlying MARCO‐mediated suppression of the NLRC5‐MHC‐I axis, the assay for transposase‐accessible chromatin with sequencing (ATAC‐seq) analysis of MARCO‐knockdown (siMARCO) and control (siNC) TAMs was performed to assess genome‐wide DNA accessibility changes and directly measure chromatin remodeling activity (Table S5, Supporting Information).^[^ ^62^ ^]^ MARCO‐knockdown and control TAMs showed a significant difference in a major portion of the chromatin accessibility peaks. The effects of MARCO on the whole chromatin structure in TAMs were evaluated by counting the total number of ATAC‐seq peaks in each group. Remarkably, the knockdown of MARCO resulted in the alteration in 8090 and 8943 specific peaks in two independent experiments, suggesting that MARCO has a significant effect on chromatin accessibility (Figure 6H; and Table S5, Supporting Information). KEGG analyses demonstrated that ubiquitin‐mediated proteolysis is the most significant pathway impacted by MARCO knockdown in TAMs (Figure 6I). Integrated analysis of the ATAC‐seq and RNA‐seq profiling identified two genes (*Socs1* and *Ube2l6*) that are downregulated in MARCO‐knockdown TAMs (Figure 6J,K). In accordance with the changes in ATAC‐seq peaks, a decrease in *Socs1* and *Ube2l6* mRNA levels was observed in cells from siMARCO group (Figure 6L). However, further RT‐qPCR analyses showed that only a significant decrease was observed in *Socs1* in MARCO‐knockdown TAMs (*p* < 0.0001, Figure 6M). Collectively, the ATAC‐seq analyses revealed that MARCO knockdown brought substantial chromatin remodeling in TAMs, which further proposed *Socs1* as the potential downstream gene of MARCO's regulatory network.

As an essential component of the SOCS family, SOCS1 plays a key role in both the innate and adaptive immune responses and is involved in a number of cytokine signal transductions and immune cell differentiation.^[^ ^63^ ^]^ Moreover, elevated SOCS1 expression could facilitate macrophage differentiation through promoting macrophage M2 polarization.^[^ ^64^, ^65^ ^]^ Western blot analysis also exhibited a significantly lowered SOCS1 protein level in MARCO‐knockdown TAMs (Figure 6N). According to previous studies, SOCS1 is a negative regulator that inhibits the signaling cascade mediated by JAK–STAT.^[^ ^66^ ^]^ We further sorted MARCO+ and MARCO‐ TAMs from RCC tumor tissues and performed transcriptome analyses (Figure S8, Supporting Information). The results revealed that the JAK–STAT pathways were enriched in MARCO+ TAMs (Figure S8A–C, Supporting Information). Among all JAK‐STAT family genes, only JAK1 and STAT1 differed significantly (Figure S8D–G, Supporting Information); thus, we hypothesized that MARCO might downregulate the JAK1‐STAT1 pathway via SOCS1. The expression and transcription factor activity of STAT1 were also significantly downregulated in the single‐cell atlas (Figure S8H–M, Supporting Information). The SOCS1 knockdown assays in TAMs revealed that SOCS1 knockdown would not influence the levels of JAK1 or STAT1, but would significantly upregulate the levels of phosphorylated STAT1 (p‐STAT1) and NLRC5 (Figure 6O; Figure S8N, Supporting Information). We also directly examined the role of MARCO on the subsequent changes in SOCS1 and STAT1/NLRC5 phosphorylation/expression. The MARCO knockdown in TAMs led to a significant downregulation of SOCS1, and a significant upregulation of p‐STAT1 and NLRC5 (Figure 6P; Figure S8O, Supporting Information). By acting as a pseudosubstrate for JAKs, SOCS1's kinase inhibitory domain prevents the JAK1 protein from performing its kinase activity.^[^ ^67^ ^]^ To elucidate the molecular mechanism by which SOCS1 hinders JAK1/STAT1 signaling, we examined the formation of SOCS1‐JAK1 complexes. Using co‐immunoprecipitation in normal conditions, endogenous SOCS1 was found to coprecipitate with endogenous JAK1 in TAMs (Figure 6Q–S). We further explored the mediator by which MARCO regulates SOCS1 (Figure S10, Supporting Information). We extracted the ATAC‐seq peaks in SCOS1 and performed transcription factors enrichment analyses (Figure S10A, Supporting Information), browsed the regulatory transcription factors for SOCS1 from hTFtarget database (Figure S10B, Supporting Information), and inferred the upregulated transcription factors in MARCO+ TAMs (Figure S10C, Supporting Information). The intersection of the three analyses revealed that SPI1 might be the regulatory transcription factor of SOCS1 via MARCO‐SPI1‐SOCS1 axis (Figure S10D,E, Supporting Information). To confirm this interaction, we adopted the ATAC‐seq data of SPI1,^[^ ^68^ ^]^ exhibiting up‐regulated peaks in the region of SOCS1, which could be inhibited by the SPI1 blockades (Figure S10F, Supporting Information).

Collectively, we concluded that MARCO could impair the tumor‐recognition and antigen‐presentation capacity of TAMs via the down‐regulating MHC‐I molecules, which is achieved by the SOCS1‐mediated blocking of JAK1 kinase function, thereby facilitating the inhibition of JAK1‐STAT1 pathway and downstream NLRC5‐MHC‐I pathway. This research clarifies a MARCO‐SOCS1‐mediated immunoevasive mechanism in the RCC tumor microenvironment.

### 2.7 MARCO Blockade Augments the Anti‐Tumor Immune Response and Potentiates PD‐1 Blockade Therapy in Renal Cancer Model

**Figure 7:**
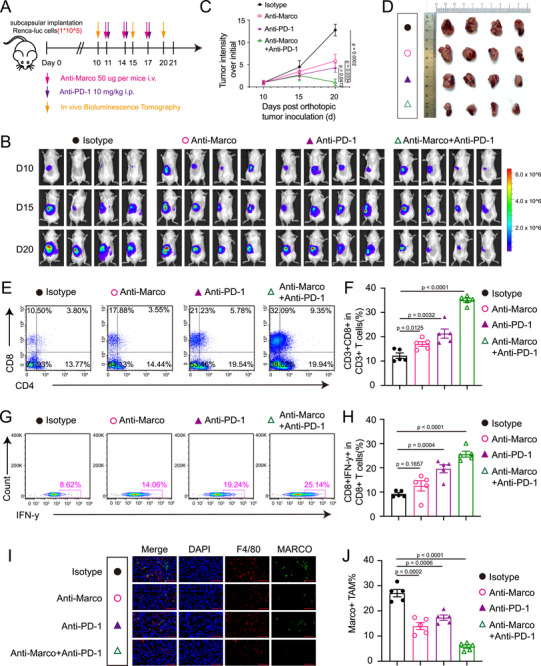
MARCO blockade enhances anti‐PD‐1‐induced tumor regression. A) The experimental design of the renal cancer tumorigenesis model and different strategies of treatment. The male mice were treated with either anti‐MARCO antibody (50 µg per mouse) with or without PD‐1 antibody (10 mg kg^−1^ body weight) as indicated starting from day 11. B) Representative In Vivo Imaging Systems images of orthotopic renal tumors of Renca‐luc cells. C) Growth curves of orthotopic renal tumors in the different treatment groups. *n* = 4 per group. Data are presented as mean ± SEM. D) General view of the kidneys of mice in the four groups. E–H) Representative flow cytometry plots and statistical analysis of the ratios of tumor‐infiltrating CD8+ and IFN‐γ+ CD8+ T cells in different treatment groups on day 21. *n* = 5 in each group. Data are presented as mean ± SEM. I) Representative multiplex immunofluorescence images staining for macrophages (F4/80, red) and MARCO (green) in the tumor tissues. *n* = 5. Scale bar, 50 µm. J) Quantification of percentages of MARCO+ macrophages in different treatment groups. *n* = 5. Data are presented as mean ± SEM. The unpaired two‐sided Student's t test was used for (C), (F), (H), and (J).

Given that MARCO+ TAMs suppress antitumor immunity of CD8+ CTLs by impairing tumor recognition, we hypothesized that restoring the antigen presentation process by MARCO blockade would likewise recover the tumor recognition by eliciting anti‐tumor activity and boost ICB therapeutic efficacy. As the treatment scheme depicted in **Figure** **7**A, we administered isotype mAbs, anti‐MARCO, anti‐PD‐1, or a combination of anti‐MARCO and anti‐PD‐1 mAbs to orthotopic Renca tumor‐bearing mice, followed by In Vivo Imaging Systems (IVIS) to evaluate the therapeutic effects on the tumor growth. Tumor volume analyses demonstrated that single anti‐PD‐1 mAbs showed moderate therapeutic effects; but when anti‐MARCO antibody was combined with anti‐PD‐1 therapy treatment, it might dramatically slow tumor development (Figure 7B). Of the tumors treated with a combination therapy, the quantitative bioluminescence intensity was only 8.0% of the control group and 23.7% of the PD‐1 mAb group (Figure 7C). Thus, the combination therapy outperformed the effects of anti‐PD‐1 agents, showcasing the superior efficacy of anti‐MARCO treatments in facilitating ICB therapies.

To assess the anti‐tumor efficacy of MARCO blockade in human RCC, we also established a patient‐derived xenograft (PDX) model. As the treatment scheme depicted in Figure S11A (Supporting Information), when the average tumor volume reached ≈100 mm^3^, mice were randomized into 4 groups and treated with isotype mAbs, anti‐MARCO, anti‐PD‐1, or combination. After 46 days of observation, tumors in the combination group were significantly smaller than in the anti‐PD‐1 group (*p* = 0.018 for tumor volume and *p* = 0.045 for tumor weight; Figure S11B–D, Supporting Information).

We further employed flow cytometry to examine the immune cells that infiltrated the RCC models following anti‐MARCO and immunotherapy‐combined treatments (Figure S11E, Supporting Information). Flow cytometry analysis revealed that the tumors treated with anti‐MARCO agents exhibited an increased proportion of infiltrated CD45+ leukocytes (Figure S12A, Supporting Information), CD3+ T cells (Figure S12B, Supporting Information), CD8+ T cells (Figure 7E,F) and IFN‐γ+CD8+ T cells (Figure 7G,H), in line with anti‐PD‐1 therapies. Anti‐MARCO antibody‐treated tumors also exhibited 2.9 times higher population of NK cells than control tumors (Figure S12C, Supporting Information). Then, for each of the four treatment conditions, we evaluated TAMs’ pro‐ and anti‐inflammatory features. Macrophages showed the greatest expression of the co‐stimulatory surface marker CD86 in mice who received combo treatment (Figure S12D, Supporting Information). In the meantime, the proportion of CD206+ macrophages in the mice's tumor tissues that received combination treatment was decreased by 12.85% (Figure S12E, Supporting Information), thus diminishing immunosuppressive effects on promoting tumor development and progression. IF staining revealed that anti‐MARCO treatment and its combination with anti‐PD‐1 mAbs significantly decreased the frequencies of MARCO+ TAMs (Figure 7I,J). Thus, combination treatment induces pronounced effects on CD8+ CTLs and TAMs in the TME, including proportional changes of IFN‐γ+ CD8+ T cells and MARCO+ TAMs as well as phenotypic alterations in TAMs toward a pro‐inflammatory phenotype. No significant changes in the population of neutrophils (CD45+CD3‐CD11b+Ly6G+), B cells (CD45+CD3‐CD19+) or Tregs (CD45+CD3+CD4+FOXP3+) were observed in the tumor TME following anti‐MARCO treatment or its combination with PD‐1 mAbs (Figure S12G–I, Supporting Information).

Together, these data suggest that pharmacologically blocking MARCO could recover the tumor recognition capacity of TAMs and boost the efficiency of ICB treatment in RCC, making it a promising therapeutic strategy for the treatment of RCC, especially for the ICB‐resistant tumors.

## 3 Discussion

Clinical research has demonstrated the significant potential of immunotherapy approaches that use checkpoint inhibitors like PD‐1 and CTLA‐4 to reverse the negative regulation of T cells.^[^ ^69^ ^]^ Single‐cell transcriptome sequencing appears to be a promising tool for the discovery of mechanisms related with ICB response.^[^ ^70^, ^71^ ^]^ In this study, to examine immune system and tumor reactions to immunotherapy, we analyzed single‐cell RNA‐seq data and comprehensively deciphered the extensive diversity in immune infiltration across ICB response‐differed RCC patients, which illustrated a significantly altered ecosystem facilitating immune escape, characterized by concentrated MARCO+ TAMs as well as limited CD8+ T cell cytotoxicity. We also identified MARCO as TAM‐enriched inhibitor of neoantigen cross‐presentation in ICB‐resistant RCCs, and demonstrated the therapeutic efficacy of targeting MARCO to recover tumor recognition and generate effective antitumor immunity in the ICB non‐responsive malignancies otherwise. Our research sheds light on the mechanisms by which MARCO governs MHC‐I molecules to alter TAMs' capacity for antigen cross‐presentation, suggesting a potential therapeutic target for RCC by modulating tumor‐associated macrophage reprogramming and restoring immune surveillance.

One critical observation from this study was the significantly remodelled CD8+ T cells activation and development in ICB‐resistant RCCs mediated by MARCO+ TAMs, resulting in significant reduction of tumor‐killing GZMK+CD8+ Tem cells as well as accumulation of tumor‐resident ZNF683+CD8+ Trm cells. ZNF683+CD8+ T cells have been described as a specific T cell population that responds to anti‐PD‐1 treatments and expresses genes linked to cytotoxicity, tissue resident memory, and T cell exhaustion.^[^ ^72^, ^73^ ^]^ The source of tumor‐infiltrating CD8+ T cells in this study has also been identified as ZNF683+ CD8+ T cells, which have the potential to become activated and develop into effector T cells. Other investigations also revealed a connection between ICB resistance and the dangling activation of CD8+ Trm cells in malignant tissues.^[^ ^74^, ^75^ ^]^ Our results suggested that ZNF683+ CD8+ T cells resident in RCC modelled by the limited tumor recognition restrained by MARCO response poorly to ICB therapies. For efficient immune detection, neoantigens generated from non‐synonymous DNA base changes must first be shown on MHC‐I or MHC‐II molecules, followed by recognition by peptide‐specific T cell receptors (TCRs).^[^ ^76^, ^77^ ^]^ Taken together, we concluded that sufficient delivery of tumor neoantigens to CD8+ Trm cells to initiate its development into anti‐tumoral effector T cells is essential for the ICB response.

Among TAMs, we identified a subset of MARCO‐expressing TAMs with immunosuppressive phenotypes in ICB non‐responders. While MARCO expression TAMs has been linked to poor prognosis in several types of solid tumors,^[^ ^44^, ^46^ ^]^ its functional significance in RCC, particularly regarding TAMs behavior and the mechanisms underlying immunotherapy resistance, remains uncharted. To investigate the cellular and molecular drivers of immunotherapy resistance, we further performed single‐cell analysis on immune cells in the TME based on the MARCO‐expressing TAMs transcriptome. The results of the analysis showed that low expression in CD8+ T cells and DCs was linked to a high abundance of MARCO+ TAMs in renal cancer tissue. This suggests that MARCO+ TAMs‐dominated RCC tissue represent an immunosuppressive microenvironment featured by low effector CD8+ T cell infiltration, undermining adaptive antitumor immunity.

Moreover, post‐ICB remaining tumor cells have markedly elevated glycolysis/gluconeogenesis and amino acid metabolism pathways. The primary process that produces lactate is glycolysis, which breaks down into hydrogen (H+) and lactate ions. Lactate ions affect CD8+ T cell anti‐tumor immune response and contribute to ICB resistance by both boosting tumor growth and suppressing CD8+ T cell function.^[^ ^78^, ^79^ ^]^ Stromal cell clustering reveals how FLT1+ ECs, which were angiogenic ECs, infiltrated ICB‐resistant tumors. FLT1 has limited kinase activity but exhibits proangiogenic tyrosine kinase activity alongside its suppressive function facilitated by the physiological splice variant.^[^ ^80^ ^]^ These FLT1+ ECs influence the TME through tumor microvessel formation and consequently cause resistance to ICB.^[^ ^81^ ^]^

We also found that the T‐effector signature and progenitor exhausted signature were strongly enriched in CD8+ T cells of low MARCO+ TAMs infiltrated TME. In responders, a specific subset of CD8+ T cells undergoes robust activation and differentiation, culminating in a terminally exhausted phenotype. Studies in melanoma models have demonstrated that the frequency of a progenitor exhausted CD8+ T cell population, which persists long‐term, responds to anti‐PD‐1 therapy, and ultimately differentiates into terminally exhausted cells within tumor‐infiltrating T cells, anticipates favorable clinical results for melanoma treated with ICB.^[^ ^11^, ^82^ ^]^ This analysis provided insight into the patterns of TAM and CD8+ T cell infiltration that may underlie treatment resistance.

Using renal tumor cell‐conditioned macrophages, we demonstrated that macrophages expressing the scavenger receptor MARCO adopt an immunosuppressive phenotype. Given that MARCO targeting restored the cytotoxicity of CD8+T cells, these suppressive macrophages were effective in preventing the proliferation of cytotoxic T cells and the generation of cytokines. Despite the fact that MARCO has already been found to be a marker for pro‐tumorigenic TAMs in NSCLC,^[^ ^45^ ^]^ breast cancer,^[^ ^52^ ^]^ and GBM,^[^ ^44^ ^]^ its role in RCC has not been thoroughly documented previously. Our findings, which is the first to associate MARCO with pro‐tumorigenic TAM traits in RCC, further underscore its significance in cancer.

Mechanistically, we observed that MARCO‐knockdown TAMs favors an activating antigen presentation and processing pathway and costimulatory molecule profile. It has been shown that macrophages use a typically necessary two‐signal procedure to activate naïve CD8+ T cells.^[^ ^83^ ^]^ Initially, naïve CD8+ T cells use their TCR complex to identify MHC‐I‐presented antigens on macrophages. Second, the major signal transduced by the TCR is modulated by costimulatory molecules of the B7 family (e.g., CD80, CD86, and B7‐H1) expressed on macrophages interacting with their equivalent receptors (e.g., CD28 and PD‐1) on T cells.^[^ ^84^ ^]^ In the presence of MARCO knockdown TAMs, we demonstrated that inhibiting MHC‐I molecules reduced CD8+ T‐cell antitumor immunity. Besides, MARCO knockdown TAMs showed enhanced NOD‐like receptor signaling, with upregulated NLRs family genes (e.g., *Nod1*, *Nod2*, *Nlrc5*) compared to control TAMs. Previous data showed that NLRC5 translocated to the nucleus, where it assembled with the RFX‐complex, ATF1/CREB, and the NFY‐complex (which bound to the SXY‐module) to form an MHC‐I enhanceosome, thereby activating the transcription of MHC‐I.^[^ ^84^, ^85^ ^]^ Our data expand on these modes of regulation in renal tumor cell‐conditioned macrophages. We revealed that knockdown of NLRC5 in MARCO‐knockdown TAMs abolished the increase in MHC‐I expression observed in MARCO‐knockdown TAMs in vitro. Notably, further research is needed to determine whether particular downstream genes of MARCO are involved in the suppressive behavior that underlies the NLRC5‐MHC‐I axis in TAMs. Furthermore, we discovered SOCS1 might be the mediator of NLRC5‐MHC‐I upregulation in MARCO‐knockdown TAMs. As JAK1 pseudosubstrate inhibitors, SOCS1 proteins prevent the JAK1 catalytic domain from interacting with its STAT1 protein substrates, thereby stopping signal propagation.^[^ ^86^ ^]^ We identified a chromatin remodeling axis whereby downregulated SOCS1 induces phosphorylation of STAT1 in TAMs, resulting in NLRC5 expression. Thus, MARCO itself can intrinsically impair the antigen presentation and processing within TAMs by deactivating the SOCS1/JAK1/STAT1/NLRC5 signaling cascade, which can decrease MHC‐I expression on the surface of TAMs, thereby forming TAMs' immunosuppressive characteristics.

Immune checkpoint drugs have increased patient survival in clinical settings and transformed the treatment of cancer. However, resistance can develop fast in cancer patients, thus only around 30% of patients benefit from immune checkpoint treatment.^[^ ^87^, ^88^ ^]^ Combination therapies generally yield superior outcomes compared to monotherapy, and this principle also applies to cancer immunotherapy based on checkpoint blockade. Based on our results here, MARCO shows potential as a therapeutic strategy to improve antigen presentation and enhance the innate‐driven antitumor response in RCC. Our findings are consistent with prior studies^[^ ^52^, ^89^ ^]^ that demonstrated MARCO blockade improved an anti‐tumor effect mediated by anti‐PD‐1 therapy. These findings also offer a promising therapeutic strategy to overcome ICB resistance and extend the applicability of cancer immunotherapy to diverse tumor types, including traditionally refractory “cold” tumors. Recently, a humanized anti‐MARCO monoclonal antibody (mAb), termed PY265,^[^ ^90^ ^]^ entered a first‐in‐human phase I trial (NCT05560191). The study is enrolling patients with advanced or metastatic solid tumors to evaluate single‐agent safety and preliminary activity, followed by combination with a PD‐1 inhibitor. Our faith in the potent therapeutic potential of this strategy is strengthened by the preclinical and nonclinical evidence that PY265 immunotherapy, either by itself or in combination with an ICB, has enhanced both the overall response and the durability of response. Overall, this trial is a significant step toward investigating new options for cancer patients, and we are eagerly awaiting the ongoing trial's findings.

There are some limitations in this study. First, the discovery of MARCO+ TAMs as a major facilitator in RCC immune escape and immunotherapy resistance were based on a small number of patients. The relatively small size of the patients and the lack of data for some analyses may raise the potential for patient‐specific biases. Future validation will be required on other immunotherapy cohorts. To address this problem, we have repeatedly verified the results in subsequent experiments and validated the prognostic effects of MARCO+ TAMs in large ICB cohort. Second, therapeutic efficiency of MARCO blockades needs further validation. Although it was hard for us to validate the therapeutic efficiency of MARCO blockades in human participants during the preclinical phase, we have established mice models, including both subcutaneous and orthotopic models, to examine whether MARCO blockades could improve the therapeutic efficiency of ICB agents. Lastly, although we have validated the results in perspective cohort, most of the clinical data in this study were based on retrospective, descriptive design. Therefore, more perspective studies were needed to validate our findings.

In conclusion, we established a large‐scale single‐cell dataset with 103345 single‐cell transcriptomes from ICB‐treated RCC patients, systematically investigating the cellular and molecular alterations related to immunotherapy resistance. This single‐cell immunotherapy atlas illustrates an immunosuppressive ecosystem in ICB‐resistant patients, with preferential infiltration of MARCO+ TAMs and restrained cytotoxicity of CD8+ T cells. Further analyses reveal that MARCO+ TAMs are related with enhanced immune escape by disrupting the development of CD8+ CTLs, whereas MARCO knockdown recovers the tumor‐recognizing functions of TAMs by enhancing the antigen cross‐presenting capacity via MHC‐I molecules, thereafter activating CD8+ CTLs. Mechanistically, MARCO induces activation of SOCS1, thereby downregulating MHC‐I expression through inhibition of the JAK1/STAT1/NLRC5 signaling cascade. MARCO blockade significantly boosts ICB's benefits in in vivo models by recovering immune recognition of tumor cells and enhancing CD8+ CTL infiltration and effector function. Taken together, our research demonstrates that MARCO blockade may be used as a tactic to increase the efficacy of treatments that target the adaptive branch of the immune response, supporting and expanding the application of immune checkpoint therapy in RCC.

## 4 Experimental Section

### Data and Software Availability

The curated single‐cell data was deposited in the Mendeley Repository (https://data.mendeley.com/datasets/mh6wb9k9f3). The scRNA‐seq source data of patients receiving ICB treatment in this study could be sourced from Sequence Read Archive (SRA) (PRJNA705464) and dbGaP (phs002065.v1.p1) databases.^[^ ^75^, ^91^ ^]^ Bulk transcriptomic expression data and clinical information from TCGA‐KIRC cohort were retrieved from XENA database (http://xenabrowser.net/datapages/).^[^ ^92^, ^93^ ^]^ The PD‐1 blockade dataset of advanced RCC was derived from Braun et al.^[^ ^94^ ^]^ The mice tumor RNA‐seq and ATAC‐seq data were provided in the Supporting Information. Further data details can be obtained from the corresponding author upon reasonable request.

### Single‐Cell RNA‐seq Data Integrating and Processing

The single‐cell transcriptome data of patients receiving ICB treatments, including ICB treatment‐naïve tumor samples, ICB‐resistant samples (post treatments) and ICB‐sensitive samples for the investigation of ICB‐resistant mechanisms was collected. Besides, adjacent normal tissues and PBMC samples were also included for comparison. Taken together, a total of 6 adjacent normal, 9 ICB treatment‐naïve tumor, 5 ICB‐sensitive tumor, 4 ICB‐resistant tumor, and 2 PBMC samples were obtained.

The *Cell Ranger* software (Version 7.0.0) was used with default settings to align and quantify single‐cell transcriptome data in FASTQ format published by 10x Genomics against the GRCh38 human reference genome.^[^ ^95^ ^]^ The *Cell Ranger* software's quantified count matrix was loaded into the *Seurat* tool (Version 4.1.1) for further analyses.^[^ ^96^ ^]^The cells were then subjected to quality control. Basically, cells with fewer than 500 identified genes and those with greater than 10% mitochondrial content were eliminated. To further exclude probable doublets, cells containing more than 8000 identified genes were discarded. Possible doublets predicted by the *DoubletFinder* software were eliminated so as not to impede the analyses.^[^ ^97^ ^]^ After filtering, samples containing fewer than 500 cells were deemed of poor quality and eliminated. More than 1000 thousand cells of good quality were preserved for further analysis. All individual high‐quality single‐cell samples were then curated into a single object and to eliminate batch effects. The dimensionality of this dataset was reduced through principal component analysis (PCA) with highly variable features, and the first 15 PCs were selected for investigation. Then, unsupervised clustering was approximated using the shared nearest‐neighbor network produced by the Louvain algorithm and the edge weights between any two cells. Using the t‐Distributed Stochastic Neighbor Embedding and Uniform Manifold Approximation and Projection (UMAP) methods, the identified clusters were displayed. The differentially expressed markers of the resultant clusters were identified and used the default nonparametric test, the Wilcoxon rank sum test with Bonferroni correction, to label the cell clusters.

### Cell–Cell Interaction Analysis

The CellChat program was utilized to study cell–cell interactions between various TME components.^[^ ^98^ ^]^ The input files for the statistical analysis function consisted of a raw count matrix and the relevant annotation file of cell types, both derived from the Seurat object. On the basis of their average expression, a visualization of the probable interaction intensity between ligand and receptor was anticipated. For additional demonstration, significant ligand‐receptor pairings were extracted.

### Single‐Cell Trajectory Analysis

The *Monocle3* algorithm was used to infer the interconversion and evolutionary paths to examine the plasticity and dynamic differentiation of individual cells.^[^ ^99^ ^]^ To reflect the intrinsic physiological properties of the cells, an unsupervised method was choosed. The clusters were specifically segregated into sizable, clearly defined divisions, and a principal graph was fitted for each partition. Then, the principal graph was learned with the Euclidean distance ratio set to 0.1, minimal branch length set to 10 and geodesic distance ratio set to 0.8 to construct the cell trajectories.

### Differential Abundance Analysis

Differences in cell abundance within specific neighborhoods between two conditions (such as tumor vs normal) were tested for using Milo (v1.2.0).^[^ ^100^ ^]^ A K‐Nearest Neighbors (KNN) graph with k = 30 was originally built using 30 primary components. Subsequently, cells were assigned to neighborhoods on the basis of their connectivity over the KNN graph. In order to test for differential abundance, Milo uses the quasi‐likelihood F‐test with a given contrast to calculate a *p*‐value for each neighborhood after fitting an negative binomial generalized linear model to the counts for each neighborhood and taking into account the varying numbers of cells between samples using TMM normalization. A 10% spatial false discovery rate (FDR) was used as a significant level and the spatial FDR built into *Milo* to account for repeated testing. For visualization, the spatial FDR and log2‐fold change of the number of cells in each neighborhood between two conditions were utilized.

Besides, another method of *Ro/e* approach was utilized to assess the enrichment or depletion of each cell subset in specific tissue types.^[^ ^28^, ^38^ ^]^ Briefly, the ratio of the observed cell number was calculated to the random expectation using a chi‐square test for each cluster across different tissue groups. A *Ro/e* value > 1 indicates enrichment, while a *Ro/e* score < 1 signifies that the cells in a particular tissue or subtype were being depleted.

### Analyses of Spatial Transcriptome Data

The 10X Visium spatial transcriptome dateset of RCC tumors is available from GEO database under accession code of GSE175540.^[^ ^53^ ^]^ Filtered barcode counts data coupled with spatial information were imported into the *Seurat* package. For each sample, corrected raw counts were normalized using *SCTransform*. The data was then merged and processed to dimensionality reduction using PCA, followed by a UMAP dimensional reduction using 30 principal components. Than cell‐type labels were transferred from the well‐annotated scRNA‐seq data of RCC atlas to newly generated spatial transcriptome data. Finally, prediction scores were got for each spot for each class.

### Bulk Transcriptomic Expression Data of Human Samples

Bulk transcriptomic expression data and clinical information from TCGA database were retrieved from XENA database (http://xenabrowser.net/datapages/).^[^ ^92^, ^93^ ^]^ The PD‐1 blockade dataset of advanced RCC was derived from Braun et al.^[^ ^94^ ^]^

### Enrichment Analysis

The *clusterProfiler* package (Version 4) was used with default parameters to perform GSEA analyses based on GO and KEGG pathways on the differentially expressed markers.^[^ ^101^ ^]^ Single sample gene set enrichment analysis (*ssGSVA*) algorithm, a nonparametric and unsupervised algorithm from the *GSVA* package (Version 1.14.1)^[^ ^102^ ^]^ was chosen to evaluate the enrichment scores for specific cell clusters in single‐cell datasets and bulk transcriptome datasets.

### Survival Analysis

For analysis and visualization, the *Survival* (Version 2.42–3) and *Survminer* (Version 0.4.9) packages were utilized. The best cut‐off value for survival analysis inferred by *Survminer* packages was used to stratify the expression levels of genes or the *ssGSVA* scores of a gene set.

### Cell Culture and Transfection

Renca (mouse kidney cancer cell) were acquired from American Type Culture Collection. In a humidified incubator (Thermo Scientific) with 5% CO_2_, Renca were cultivated in RPMI 1640 (Gibco), supplemented with 10% fetal bovine serum (FBS, Gibco) and 1% penicillin/streptomycin solution (Gibco) at 37 °C. For knockdown assays, the MARCO siRNA and negative control siRNA (Genepharma) were transfected into the BMDMs at 25 pmol using Lipofectamine RNAiMAX Transfection Reagent (13778150, Invitrogen).

### Human RCC Samples

Paraffin‐fixed kidney tissue from advanced RCC patients who received immunotherapy were collected in the institute. Every sample was taken with the Ethics Committee of Peking University Cancer Hospital's approval (2023KT58) and with the informed consent of the patient.

### Immunofluorescence and Image Analysis

4‐um‐thick slices were fixed in cold acetone for 3 min and removed from the blood using perfusion of 50 mL of phosphate‐buffered saline (PBS). Tissue sections were blocked with 1% bovine serum in PBS at room temperature for 30 min prior to staining. After being incubated at 4 °C for the whole night and initially stained with a primary antibody at a strength of 1:100, the sections were stained with secondary antibodies for 1 h at room temperature. The sections were then treated with the matching secondary antibody conjugated with iF 488 and iF 555. The main antibodies listed below were employed: MARCO (DF15505, Affinity), CD68 (GB115723, Servicebio), F4/80 (GB113373, Servicebio), CD8 (GB114123, Servicebio), GZMK (GB112190, Servicebio), TREM2 (A10482, Abclonal), TGFB (GB115750, Servicebio), and secondary antibodies (GB23204, GB23301, GB23302, GB23303, and GB2340, Servicebio). Following each incubation, PBS was used to wash the tissue sections. Invitrogen's Prolong Diamond mounting media was used to mount the samples. Image‐Pro Plus 6.0 and ImageJ software were used to gather and analyze the images.

### Macrophage Isolation and Priming

Bone marrow was extracted from the femurs and tibias of healthy 6–8‐week‐old BALB/c mice (SPF Biotechnology) by flushing with DMEM in order to produce BMDMs in vitro.^[^ ^103^ ^]^ Red blood cells were lysed after collection, and the leftover cells underwent two PBS washes. To induce macrophage differentiation, the cells were resuspended in Dulbecco's Modified Eagle Medium (DMEM) supplemented with 20% FBS and 20% conditioned medium containing macrophage colony‐stimulating factor (M‐CSF), which was collected from 3‐4‐day cultures of mouse L929 fibroblasts secreting M‐CSF. The cells were plated in different‐sized containers according to the planned use. On day 3, half of the culture media was supplemented with fresh DMEM/M‐CSF, and by Day 6, fresh complete mixture was supplemented. Mature BMDMs were obtained after 10 days of induction. Alternatively, utilizing transwell inserts to prevent any direct cell‐to‐cell contact, macrophages that had differentiated over a 10‐day period were cocultured with the renal cancer cell line Renca for 48 h. Additionally, just 10% FBS was added to DMEM for M0. After stimulation, the cells underwent phenotyping and were subsequently utilized in various assays.

### RT‐qPCR

RNA was extracted from macrophages using the SteadyPure Universal RNA Extraction Kit II (Accurate Biology) in order to quantify gene expression. Using the ReverTra AceTM qPCR RT Kit (Toyobo), cDNA was reverse transcribed at 37 °C for 15 min and 98 °C for 5 min. RT‐qPCR reactions were performed using SYBR Green Realtime PCR Master Mix (Toyobo). Primers used for analysis of gene expression and mRNA quantification were for *marco, il10, cx3cr1, arg1, csf1r, cd200r, ccr2, siglec1, tgfb, cd163, nos2, il12b, h2‐ab1, nod1, nod2, nlrc3, nlrc4, nlrc5, naip1, naip2*, and *gapdh* (Tianyi Huiyuan). The reaction for cDNA synthesis was conducted in the Applied Biosystems Veriti Thermal Cycler (Veriti Thermal Cycler, 96‐well Fast). PrimerBank provided the primer sequences. Each sample underwent triple RT‐qPCR reactions using Applied Biosystem's Fast SYBR Green, which were conducted on the Applied Biosystems 7500 Real Time PCR System. To verify the amplification specificity, melting curves were examined. The Livak method (2^−ΔΔC T^) was used to perform relative quantifications of gene expression. *Gapdh* served as the reference gene in this study.

### Bulk RNA‐sequencing of Mice Tumors

RNA was extracted from macrophages using the MJzol Animal RNA Isolation Kit (Majorivd) according to the manufacturer's instructions, then purified by RNAClean XP Kit (Beckman Coulter) and RNase‐Free DNase Set (QIAGEN), and finally quantified by Qubit 2.0 Fluorometer. To finish building the mRNA sequencing library, the purified total RNA is subjected to a number of processes, including mRNA separation, fragmentation, first‐strand cDNA synthesis, second‐strand cDNA synthesis, end repair, a‐tailing at the 3′ end, adaptor ligation, enrichment, and more. Dual‐end sequencing, with each end being read for 150 bp, is done using the PE150 (Pair‐end 150 bp) sequencing mode on the Illumina NovaSeq6000 sequencing platform. The obtained fastq data was then aligned and quantified to get the expression matrix using *STAR* and *RSEM* software,^[^ ^104^ ^]^ based on the GRCm38 (mm10) genome and GENCODE vM10 transcript files. Subsequently, *edgeR* package was used to calculate the differentially expressed genes.

### Western Blotting

Cells were collected and lysed right away using ice‐cold RIPA lysis buffer (P0013B, Beyotime) and protease and phosphatase inhibitors (4 693 132 001, Roche) in preparation for western blotting. Protein concentration was determined using the BCA protein estimation procedure (23 227, Thermo Fisher). On 10% SDS polyacrylamide gels (NP0315BOX, Invitrogen), equivalent quantities of protein were electrophoretically separated before being transferred to PVDF membranes (IPVH00010, Merck Millipore). Following MARCO (MCA1849, Biorad), MHC‐I (TX4203, Abmart), NLRC5 (A16740, Abclonal), β‐actin (GB15003, Servicebio), SOCS1 (55 313, CST), JAK1 (50 996, CST), STAT1 (14 994, CST), and phosphor‐STAT1 (9167, CST) being first probed on the blots, they were probed once more using secondary HRP‐conjugated anti‐rabbit (7074, CST,) or anti‐mouse (7076, CST) antibody following further washing. Enhanced chemiluminescence (ECL) was used to visualize the antigen‐antibody reaction (WBKLS, Millipore). The relative western‐blot band intensity had been quantified from at least three independent biological replicates and were normalized for each β‐actin band. Data were presented as mean ± SEM.

### CD8+ T Cell Isolation and Function Assay

A MojoSort Mouse CD8+ T Cell Isolation Kit (480 007, BioLegend) was used to magnetically separate splenic CD8+ T cells from mice in accordance with the manufacturer's instructions. The cells were then activated by CD3 (100 239, BioLegend)/CD28 (102 115, BioLegend) in a mixed lymphocyte response for a duration of 24 h. Isolated CD8+ T cells were first labeled with 10 µm carboxyfluorescein diacetate succinimide ester (CFSE, 423 801, BioLegend) and then cocultured with BMDMs at a ratio of 2:1 for 3 days. The production of interferon (IFN)‐γ, and CD8+ T‐cell proliferation were then assessed after 6 h of stimulation with PMA (100 ng mL^−1^) and ionomycin (500 ng mL^−1^) before staining and flow cytometry analysis.

### Tumor Cell Killing Assay

CSFE (423 801, BioLegend) was applied to target cells (Renca) for 20 min at 37 °C. Effector cells (CD8+ T after coculture with BMDMs) and prelabeled target cells were co‐incubated for 6 h at 37 °C with an E:T ratio of 10:1. Dead cells differentiated from live cells by staining with a Zombie Aqua Fixable Viability Kit (423 101, BioLegend).

### Animal Studies

6–8‐week‐old male Balb/c mice and 4–6‐week‐old NOD/SCID male mice were supplied by SPF (Beijing, China) Biotechnology Company Limited. Mice were maintained at the Peking University Cancer Hospital, and the trials were authorized by the Peking University Cancer Hospital (EAEC 2025‐09). In accordance with local ethical standards, mice were housed and reared in pathogen‐free environments.

For subcutaneous tumor model, 100 uL RPMI 1640 containing 4 × 10^5^ Renca cells and 2 × 10^5^ Control‐sh and MARCO^KD^ BMDM cells expressing shRNA against β2m were subcutaneously administered to these mice's right sides. Subcutaneous tumor volumes were calculated using the formula (width^2^×length)/2. Mice were monitored for tumor growth every 2 days afterward by manual measurements.

For orthotopic tumor model, mice were given anesthesia before receiving 25 µL of a single‐cell solution containing Renca‐luc (1 × 10^5^ cells) in BD Matrigel into the right kidney cortex. At days 11, 14, and 17 of the tumor inoculation, mice were injected with 50 ug of anti‐MARCO mAb (*i.v*., rat IgG1; clone ED31), 10 mg kg^−1^ mice body weight of anti‐PD‐1 mAb (*i.p*., BE0146, clone RMP1‐14, BioXcell) or IgG1 isotype mAb (*i.p*., BE0083, clone MOPC‐21, BioXcell). Utilizing IVIS, the mice were imaged on day 10, 15, and 20 of the experiment. For the operation, isoflurane was used to anesthetize the mice.

For PDX model, fresh tissues from a patient with RCC were cut into pieces and subcutaneously inoculated into mice. When the tumor volume reached ≈100 mm^3^, mice were treated with 50 ug of anti‐MARCO mAb (*i.v*., MA5‐48228, Invitrogen), 10 mg kg^−1^ mice body weight of anti‐PD‐1 mAb (*i.p*., JS‐001, Junshi Biosciences) or IgG1 isotype mAb (*i.p*., BE0297, BioXcell). Tumor volumes were calculated using the formula (width^2^×length)/2. Mice were monitored for tumor growth every 3 days afterward by manual measurements.

### Flow Cytometry Analysis

For 30 min on ice, BMDMs were stained with fluorescent conjugated antibodies in order to perform cell surface MARCO, MHC‐I, MHC‐II, CD40, CD80, and CD86 analyses. After two RPMI 1640 washes, the cells were suspended in PBS for examination.

Using surgical scalpels, Renca tumor tissues were chopped into small pieces for tumor tissue flow cytometry examination. They were then further enzymatically dissociated in RPMI 1640 with 100 µg mL^−1^ DNase I, 0.5 mg mL^−1^ hyaluronidase, and 1 mg mL^−1^ collagenase V for an hour at 37 °C. To prevent clumping, 0.1 m EDTA was added after 20 min of incubation. Following the lysis of red blood cells, cells were labeled using a Zombie Aqua Fixable Viability Kit (423 101, BioLegend) and blocked using anti‐mouse CD16/CD32 (TruStain FcX, clone 93, BioLegend). The following mouse antigens were then added to the cells and incubated with the indicated fluorescent conjugated antibodies: PerCP‐Cy5.5‐CD45 (30‐F11), AF488‐CD3 (17A2), PE/Cy7‐CD11B (M1/70), BV605‐F4/80 (BM8), APC‐CD86 (GL‐1), BV421‐CD206 (C068C2), APC/Fir750‐LY6G (1A8), PE‐NKp46 (29A1.4), PE/Cy7‐CD8 (53‐6.7), BV421‐IFN‐γ (XMG1.2), APC‐GZMB (QA16A02), AF700‐CD4 (RM4‐4), APC/Fir750‐CD19 (6D5), APC‐CD25 (PC61), and PE‐FOXP3 (MF‐14). All antibodies were purchased from BioLegend. The cells were then suspended in PBS after being fixed in 4% paraformaldehyde. The samples were analyzed using a Beckman Dmi8 cytometer and analyzed with CytExpert software.

### ATAC‐seq

Centrifugation was used to gather BMDM nuclei, and Tn5 transposases were used for tagmentation. The amplification process lasted 30 min at 37 °C. Purification and screening with 1.5×NGS magnetic beads produced the final library. *Fastp* program (version 0.23.0) was used to evaluate the libraries' quality. The reference genome was aligned with clean data to gain complete transcript information. An Illumina HiSeq XTen sequencer was used for the sequencing process. The site of the ATAC‐seq peak on the genome and the sequencing information of the peak area were obtained using MACS2. *Bedtools* and *edgeR* were used for peak annotation and distinction. After that, motif analysis was done using the HOMER program. Lastly, ATAC‐seq data analyzed together with that of RNA‐seq were used to discover DEGs that regulate chromatin accessibility.

### Co‐Immunoprecipitation

As directed by the manufacturer, immunoprecipitation experiments were carried out using an immunoprecipitation kit (88 804, Thermo). In short, the cells were lysed in RIPA buffer, and then the lysate was mixed with SOCS1 antibody (55 313, CST) and IgG (3900, CST) before being incubated at 4 °C for the entire night. To pull down the particular protein complex interacting with SOCS1, magnetic beads were introduced. The eluted complex was subjected to Western blot analysis using the JAK1 antibody (50 996, CST) and the SOCS1 antibody (55 313, CST).

### Statistical Analysis

R program 4.2 and GraphPad Prism 8 were used for statistical analysis. The results are shown as the mean ± SEM of at least three separate studies. As directed, one‐way ANOVA and the two‐tailed unpaired Student's t test or Wilcoxon test were used to examine the data. Significant differences were calculated using the log‐rank test in survival analysis. A two‐side *p*‐value of less than 0.05 was deemed statistically significant.

## Conflict of Interest

X.S. has the following potential conflicts of interest to disclose: non‐profit consultant for Pfizer, Novartis, Astellas, AstraZeneca, Bayer, MSD, RemeGen, and Junshi Biosciences. J.G. is a member of the advisory board/consultant of MSD, Roche, Pfizer, Bayer, Novartis, Simcere, Shanghai Junshi Bioscience, and Oriengene. The other authors declare no competing interests.

## Author Contributions

X.W. and X.S. performed conceptualization, supervision, and funding acquisition; J.C., X.W., and X.S. performed methodology, validation, and wrote the original draft; J.C. and J.M. performed software; J.C., J.M., J.W., M.J., J.D., Y.K., X.W., and X.S. performed formal analysis; J.D., Y.K., C.C., L.S., and Z.C. performed investigation; J.C., J.D., Y.K., X.Y., J.G., X.W., and X.S. provided resources; J.C. and X.W. performed data curation; J.C. performed visualization; All authors wrote, reviewed, and edited the draft.

## Supporting information



Supporting Information

Supplemental Table 1

Supplemental Table 2

Supplemental Table 3

Supplemental Table 4

Supplemental Table 5

## Data Availability

The curated single‐cell data was deposited in the Mendeley Repository (https://data.mendeley.com/datasets/mh6wb9k9f3). The scRNA‐seq source data of patients receiving ICB treatment in this study could be sourced from SRA database (PRJNA705464) and dbGaP (phs002065.v1.p1).^[^ ^75^, ^91^ ^]^ Bulk transcriptomic expression data and clinical information from TCGA database were retrieved from XENA database (http://xenabrowser.net/datapages/).^[^ ^92^, ^93^ ^]^ The PD‐1 blockade dataset of advanced RCC was derived from Braun et al.^[^ ^94^ ^]^ The mice tumor RNA‐seq and ATAC‐seq data were provided in the supplementary data. The 10X Visium spatial transcriptome dataset of RCC tumors is available from GEO database under accession code of GSE175540,^[^ ^53^ ^]^ the SPI1 ATAC‐seq dataset is available from GEO database under accession code of GSE236085,^[^ ^68^ ^]^ and the ICB treatment cohort of colorectal cancer dataset is available from GEO database under accession code of GSE236581.^[^ ^47^ ^]^ Further data details can be obtained from the corresponding author upon reasonable request.
