# Supplementary material for: MARCO+ Tumor‐Associated Macrophages Impede CD8+ T Cell Immunity to Facilitate Immunotherapy Resistance in Renal Cell Carcinoma
Source: Adv Sci (Weinh). 2025 Oct 21;12(47):e14600. doi: 10.1002/advs.202514600 (PMC12713061; doi:10.1002/advs.202514600)
Supplement: Supplementary file 1 — Supporting Information [file ADVS-12-e14600-s005.pdf]

MARCO+ tumor-associated macrophages impede CD8+ T cell immunity to facilitate immunotherapy resistance in renal cell carcinoma

Jiayuan Chen, Jiazhi Mo, Jinchang Wei, Mengnan Qu, Jie Dai, Yan Kong, Huayan Xu, Juan Li, Xieqiao Yan, Chuanliang Cui, Lu Si, Zhihong Chi, Jun Guo, Xiaowen Wu* and Xinan Sheng*

Supplementary Figure and Table Legend

Figure S1. Markers to define the major cell lineages in single-cell atlas of RCC patients

(A) Uniform Manifold Approximation and Projection (UMAP) embedding of scRNA-seq data by tissue types, datasets, ICB efficiency and samples. (B) UMAP embedding showing the RNA expression of marker genes used to define the 15 major cell types. (C) Stacking bar plot showing the cell distribution and heterogeneity across groups and samples.

Figure S2. Landscape of T/NK lymphocytes

(A) Dot plot showing the markers using to define the CD4+ T, CD8+ T, natural killer (NK) and NK-like T (NKT) cell clusters. (B) Uniform Manifold Approximation and Projection (UMAP) embedding and cell abundance of T/NK cells for five tissue types: adjacent normal, immune checkpoint blockade (ICB) treatment-naïve tumor (non-ICB), ICB sensitive tumor (partial response [PR]), ICB-resistant tumor (progressive or stable diseases [PD/SD]) and peripheral blood mononuclear cell (PBMC) samples. (C) Heatmap displaying the distribution of T/NK cells across different tissue types (normal, non-ICB, ICB PR, ICB PD/SD and PBMC), as estimated by Ro/e. (D) Boxplot comparing the abundances of T/NK cells across adjacent normal (n = 9) and RCC tumor tissues (n = 18). (E) Boxplot comparing the abundances of T/NK cells across ICB sensitive (n = 5) and resistant tumor tissues (n = 4). (F) Dot plot showing the results of KEGG enrichments of the differentially expressed genes of CD8+ T cells in the ICB sensitive and resistant groups. (G) Violin

plots comparing the expression of marker genes in the ICB sensitive and resistant tumor cells. The unpaired two-sided Student's t test was used for (D) and (E). Two-sided Wilcoxon test was used for (G).

Figure S3. Landscape and metabolic analyses of RCC tumor cells

(A) Uniform Manifold Approximation and Projection (UMAP) embedding of RCC tumor cells. (B) Dot plot showing the markers using to define the clusters of RCC tumor cells. (C) UMAP embedding and cell abundance of scRNA-seq data for tumor cells in immune checkpoint blockade (ICB) treatment-naïve tumor (non-ICB), ICB sensitive tumor (partial response [PR]) and ICB-resistant tumor (progressive or stable diseases [PD/SD]) tissues. (D) Dot plot showing the results of Reactome and KEGG enrichments of the differentially expressed genes in the ICB sensitive and resistant tumor cells. (F) Violin plots comparing the single sample gene set variation analysis (ssGSVA) scores of glycolysis/Gluconeogenesis and amino acid metabolism in the ICB sensitive and resistant tumor cells. Two-sided Wilcoxon test was used for (F).

Figure S4. Landscape of RCC stromal cells

(A) Uniform Manifold Approximation and Projection (UMAP) embedding of RCC stromal cells. (B) Dot plot showing the markers using to define the clusters of RCC stromal cells. (C) UMAP embedding and cell abundance of scRNA-seq data for stromal cells in adjacent normal, immune checkpoint blockade (ICB) treatment-naïve tumor (non-ICB), ICB sensitive tumor (partial response [PR]) and ICB-resistant tumor (progressive or stable diseases [PD/SD]) tissues. (D) Dot plot showing the results of GO BP (biological process) and MF (molecular function) enrichments of the differentially expressed genes in the ICB PR and ICB PD/SD stromal cells.

Figure S5. Markers to define the cell lineages of myeloid cells

(A) Heatmap showing the markers using to define the myeloid cell clusters. (B) Dot plot showing the markers using to define the myeloid cell clusters. (C) Dot plot showing the markers of M1 macrophage, M2 macrophage, angiogenesis, phagocytosis

and checkpoints in the myeloid cell clusters. (D) Comparison of the abundance of myeloid cell clusters in the pre-ICB (n = 9) and post-ICB samples (n = 9) in the RCC single-cell atlas. (E) Comparison of the abundance of myeloid cell clusters in the pre-ICB (n = 10) and post-ICB samples (n = 16) in an external cohort. The data was derived from colorectal cancer (GSE205506) reported by Li et al. The cell annotation was transferred from the RCC single-cell atlas. (F) Representative images of multiplex immunofluorescence staining of DAPI, CD68, MARCO, TREM2 and TGFB in RCC tumors. Representative experiment out of n = 3 biological replicates. Scale bar, 50 μm.

Figure S6. Cell trajectory of CD8+ T cells

(A) Cell trajectory and pseudotime of CD8+ T cells for adjacent normal, immune checkpoint blockade (ICB) treatment-naïve tumor (non-ICB), ICB sensitive tumor (partial response [PR]), ICB-resistant tumor (progressive or stable diseases [PD/SD]) and peripheral blood mononuclear cell (PBMC) tissues. (B) Expression of marker genes of CD8+ T cells by pseudotime.

Figure S7. *In situ* interaction analyses of MARCO+ TAM and CD8+ T cells

(A) *In situ* interaction analyses of MARCO+ TAM and CD8+ T cells using spatial transcriptome of ccRCC (10X Visium data, Meylan et al 2022, GSE175540). The cell-lineage specific scores were transferred from the RCC single-cell atlas. (B) Representative images of mIF comparing in situ co-localization of CD8+ T cells and MARCO+ TAMs in RCC samples. Four markers were stained: DAPI, CD68, MARCO and CD8. Representative experiment out of n = 3 biological replicates. Scale bar, 100 μm.

Figure S8. Validation of the MARCO-SOCS1-JAK1-STAT1 pathway using human RCC-derived MARCO+ TAM and MARCO- TAM

(A) Transcriptome sequencing was performed on MARCO+ TAMs (n = 3) and MARCO- TAMs (n = 3) from human RCC samples. (B) Top 5 pathways changed in

MARCO+ TAMs (n = 3) and MARCO- TAMs (n = 3) revealed by KEGG gene-set enrichment analysis (GSEA) pathway enrichment analysis. (C) GSEA results showed that JAK-STAT pathway and antigen presentation pathway were down-regulated in MARCO+ TAMs. (D) Boxplot to compare the expression of JAK family genes from MARCO+ TAMs (n = 3) and MARCO- TAMs (n = 3). (E) Boxplot to compare the expression of STAT family genes from MARCO+ TAMs (n = 3) and MARCO- TAMs (n = 3). (F) Boxplot to compare the expression of SOCS family genes from MARCO+ TAMs (n = 3) and MARCO- TAMs (n = 3). (G) Boxplot to compare the expression of MHC family genes from MARCO+ TAMs (n = 3) and MARCO- TAMs (n = 3). (H) Pseudotime and cell trajectories of tissue-resident macrophages (TRM)-derived macrophages inferred by Monocle2. (I) Expression of HLA-A and HLA-B by pseudotime in cell trajectories of TRM-derived macrophages. (J) Expression of HLA-A and HLA-B in cell trajectories of TRM-derived macrophages. (K) Expression and (L) transcription factor activity of STAT1, CEBPG and RXRA in cell trajectories of TRM-derived macrophages. (M) Dynamic changes in transcription factor activity (top) and expression (bottom) of STAT1 over time. (N) The quantitation of band intensity of SOCS1, JAK1, STAT1, p-STAT1 and NLRC5 in TAMs from siNC and siSOCS1 groups using β-Actin as loading control (n = 3). Data are presented as mean ± SEM. (O) The quantitation of band intensity of MARCO, SOCS1, JAK1, STAT1, p-STAT1 and NLRC5 in TAMs from siNC and siMARCO groups using β-Actin as loading control (n = 3). Data are presented as mean ± SEM. The unpaired two-sided Student's t test was used for (D) (E) (F) (G) (N) and (O). Two-sided Kruskal-Wallis test was used for (J) (K) and (L).

Figure S9. Representative contour plot of tumor-infiltrating (A) CD8+ T cells, (B) IFN-γ+ CD8+ T cells and (C) GZMB+ CD8+ T cells in subcutaneous RCC tumor samples as determined by flow cytometric analysis. Data are presented as mean ± SEM. The unpaired two-sided Student's t test was used for (A) (B) and (C).

Figure S10. ATAC-seq data to investigate the upstream transcript factors

mediated by MARCO to regulate SOCS1

(A) Top 10 enriched transcript factors (TFs) in the ATAC-seq peaks detected in siNC TAMs compared by siMARCO TAMs. (B) Top 10 enriched TFs for the target gene (SCOS1) in the hTFtarget databases. (C) Top 10 up-regulated TFs according to transcription factor activity in MARCO+ TAMs. (D) Intersection of the three datasets to investigate the upstream transcript factors mediated by MARCO to regulate SOCS1. (E) Dynamic changes in transcription factor activity (left) and expression (right) of SPI1 over time. (F) The ATAC-seq peaks detected in IgG, SPI1 and SPI1+SPI1 inhibitor groups. The data was derived from the GEO database (GSE236085).

Figure S11. Anti-tumor effect of MARCO blockade and anti-PD-1 combination therapies in RCC PDX model, and gating strategy of mice orthotopic tumor model

(A) Flow chart of the experimental design. The mice were treated with either anti-MARCO antibody (50 μg per mouse) with or without anti-PD-1 antibody (10 mg/kg body weight) as indicated starting from day 25. (B) Growth curves of tumors in the different treatment groups. n = 5 per group. Data are presented as mean ± SEM. (C) Tumor image of tumors in the different treatment groups. n = 5 per group. (D) Tumor weight of mice in the different treatment groups. n = 5 per group. Data are presented as mean ± SEM. (E) Gating strategy for identification of macrophages, M1-like macrophages, M2-like macrophages, NK cells, neutrophils, CD45+ leukocytes, CD3+ T cells, CD8+ T cells, IFN-γ+CD8+ T cells, CD4+ T cells and B cells. The unpaired two-sided Student's t test was used for (B) and (D).

Figure S12. Representative flow cytometry plots and statistical analysis of the ratios of immunocytes within orthotopic Renca tumors from mice in different treatment groups on day 21

Tumor-infiltrating immunocytes include (A) CD45+ leukocytes, (B) CD3+ T cells, (C) NK cells, (D) macrophages, (E) M1-like macrophages, (F) M2-like macrophages, (G) Tregs, (H) B cells, and (I) neutrophils. n = 5 in each group. Data are presented as

mean ± SEM. The unpaired two-sided Student's t test was used for (A) (B) (C) (D) (E) (F) (G) (H) and (I).

Table S1. Information of the samples included in this study.

Table S2. Top-ranked markers to annotate the cell lineages.

Table S3. Markers used to calculate the abundance of MARCO+ TAMs in bulk RNA-seq data.

Table S4. RNA-seq matrix and differentially expressed genes in siMARCO macrophages.

Table S5. Differentially expressed peaks of ATAC-seq data of siMARCO macrophages.

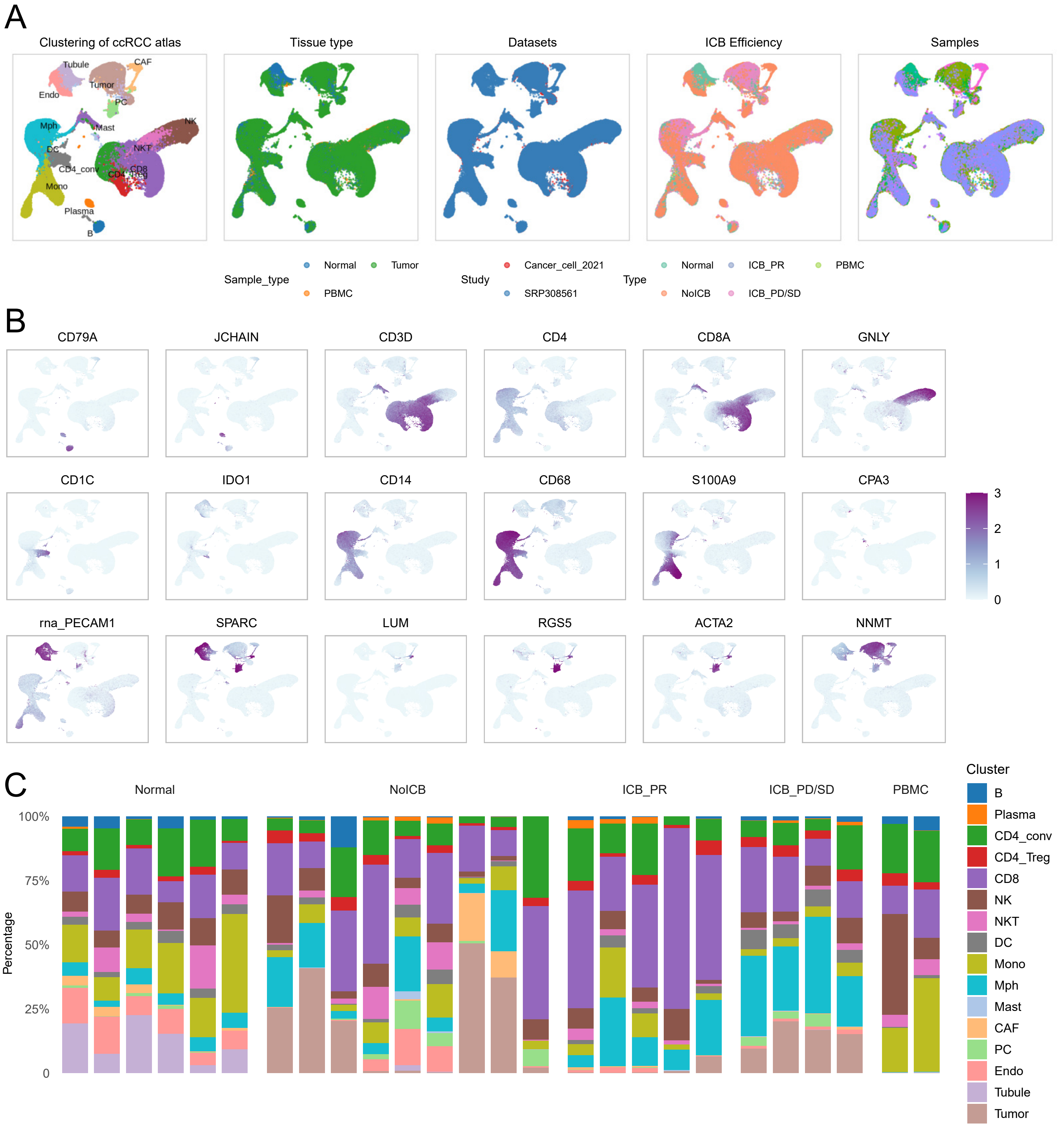
A
Clustering of ccRCC atlas
Tissue type
Datasets
ICB Efficiency
Samples
Tubule
CAF
Tumor
Endo
PC
Mph
Mast
NK
DC
NKT
CD4_conv
CD8
CD4_Treg
Mono
Plasma
B
Sample_type
Normal
Tumor
PBMC
Study
Cancer_cell_2021
SRP308561
Type
Normal
ICB_PR
PBMC
NoICB
ICB_PD/SD
B
CD79A
JCHAIN
CD3D
CD4
CD8A
GNLY
CD1C
IDO1
CD14
CD68
S100A9
CPA3
rna_PECAM1
SPARC
LUM
RGS5
ACTA2
NNMT
3
2
1
0
C
Normal
NoICB
ICB_PR
ICB_PD/SD
PBMC
Percentage
100%
75%
50%
25%
0
Cluster
B
Plasma
CD4_conv
CD4_Treg
CD8
NK
NKT
DC
Mono
Mph
Mast
CAF
PC
Endo
Tubule
Tumor

Figure S1. Markers to define the major cell lineages in single-cell atlas of RCC patients

(A) Uniform Manifold Approximation and Projection (UMAP) embedding of scRNA-seq data by tissue types, datasets, ICB efficiency and samples. (B) UMAP embedding showing the RNA expression of marker genes used to define the 15 major cell types. (C) Stacking bar plot showing the cell distribution and heterogeneity across groups and samples.

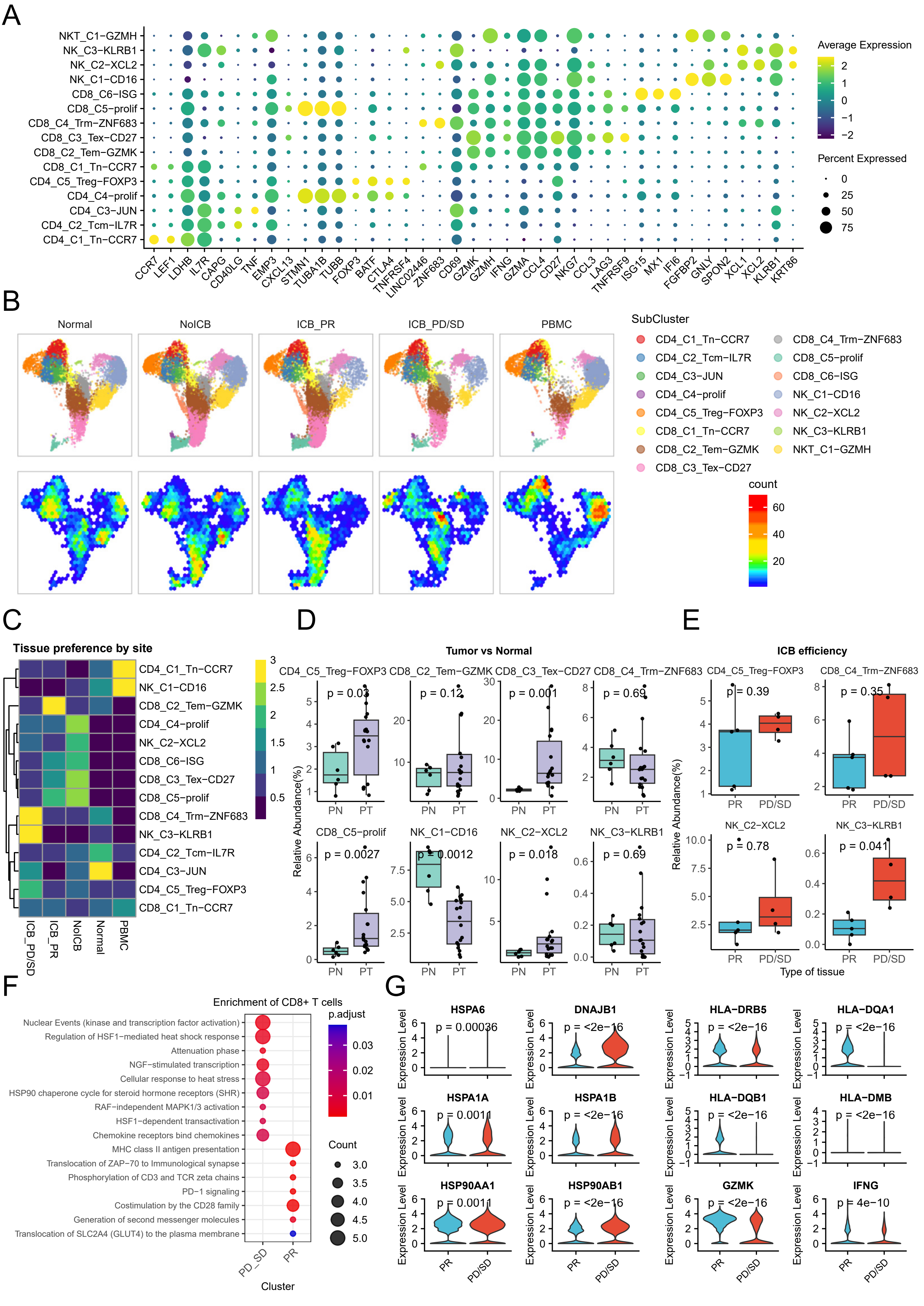
A
NKT_C1−GZMH
NK_C3−KLRB1
NK_C2−XCL2
NK_C1−CD16
CD8_C6−ISG
CD8_C5−prolif
CD8_C4_Trm−ZNF683
CD8_C3_Tex−CD27
CD8_C2_Tem−GZMK
CD8_C1_Tn−CCR7
CD4_C5_Treg−FOXP3
CD4_C4−prolif
CD4_C3−JUN
CD4_C2_Tcm−IL7R
CD4_C1_Tn−CCR7
CCR7 LEF1 LDHB IL7R CAPG CD40LG TNF EMP3 CXCL13 STMN1 TUBA1B TUBB FOXP3 BATF CTLA4 TNFRSF4 LINC02446 ZNF683 CD69 GZMK GZMH IFNG GZMA CCL4 CD27 NKG7 CCL3 LAG3 TNFRSF9 ISG15 MX1 IFI6 FGFBP2 GNLY SPON2 XCL1 XCL2 KLRB1 KRT86
Average Expression
2
1
0
−1
−2
Percent Expressed
0
25
50
75
B
Normal
NoICB
ICB_PR
ICB_PD/SD
PBMC
SubCluster
CD4_C1_Tn−CCR7
CD4_C2_Tcm−IL7R
CD4_C3−JUN
CD4_C4−prolif
CD4_C5_Treg−FOXP3
CD8_C1_Tn−CCR7
CD8_C2_Tem−GZMK
CD8_C3_Tex−CD27
CD8_C4_Trm−ZNF683
CD8_C5−prolif
CD8_C6−ISG
NK_C1−CD16
NK_C2−XCL2
NK_C3−KLRB1
NKT_C1−GZMH
count
60
40
20
C
Tissue preference by site
CD4_C1_Tn−CCR7
NK_C1−CD16
CD8_C2_Tem−GZMK
CD4_C4−prolif
NK_C2−XCL2
CD8_C6−ISG
CD8_C3_Tex−CD27
CD8_C5−prolif
CD8_C4_Trm−ZNF683
NK_C3−KLRB1
CD4_C2_Tcm−IL7R
CD4_C3−JUN
CD4_C5_Treg−FOXP3
CD8_C1_Tn−CCR7
ICB_PD/SD
ICB_PR
NoICB
Normal
PBMC
3
2.5
2
1.5
1
0.5
D
Tumor vs Normal
CD4_C5_Treg−FOXP3 p = 0.03
CD8_C2_Tem−GZMK p = 0.12
CD8_C3_Tex−CD27 p = 0.001
CD8_C4_Trm−ZNF683 p = 0.69
CD8_C5−prolif p = 0.0027
NK_C1−CD16 p = 0.0012
NK_C2−XCL2 p = 0.018
NK_C3−KLRB1 p = 0.69
PN
PT
Relative Abundance(%)
E
ICB efficiency
CD4_C5_Treg−FOXP3 p = 0.39
CD8_C4_Trm−ZNF683 p = 0.35
NK_C2−XCL2 p = 0.78
NK_C3−KLRB1 p = 0.041
PR
PD/SD
Relative Abundance(%)
Type of tissue
F
Enrichment of CD8+ T cells
Nuclear Events (kinase and transcription factor activation)
Regulation of HSF1−mediated heat shock response
Attenuation phase
NGF−stimulated transcription
Cellular response to heat stress
HSP90 chaperone cycle for steroid hormone receptors (SHR)
RAF−independent MAPK1/3 activation
HSF1−dependent transactivation
Chemokine receptors bind chemokines
MHC class II antigen presentation
Translocation of ZAP−70 to Immunological synapse
Phosphorylation of CD3 and TCR zeta chains
PD−1 signaling
Costimulation by the CD28 family
Generation of second messenger molecules
Translocation of SLC2A4 (GLUT4) to the plasma membrane
PD_SD
PR
Cluster
p.adjust
0.03
0.02
0.01
Count
3.0
3.5
4.0
4.5
5.0
G
HSPA6 p = 0.00036
DNAJB1 p = <2e−16
HLA−DRB5 p = <2e−16
HLA−DQA1 p = <2e−16
HSPA1A p = 0.0011
HSPA1B p = <2e−16
HLA−DQB1 p = <2e−16
HLA−DMB p = <2e−16
HSP90AA1 p = 0.0011
HSP90AB1 p = <2e−16
GZMK p = <2e−16
IFNG p = 4e−10
Expression Level
PR
PD/SD

Figure S2. Landscape of T/NK lymphocytes

(A) Dot plot showing the markers using to define the CD4+ T, CD8+ T, natural killer (NK) and NK-like T (NKT) cell clusters. (B) Uniform Manifold Approximation and Projection (UMAP) embedding and cell abundance of T/NK cells for five tissue types: adjacent normal, immune checkpoint blockade (ICB) treatment-naïve tumor (non-ICB), ICB sensitive tumor (partial response [PR]), ICB-resistant tumor (progressive or stable diseases [PD/SD]) and peripheral blood mononuclear cell (PBMC) samples. (C) Heatmap displaying the distribution of T/NK cells across different tissue types (normal, non-ICB, ICB PR, ICB PD/SD and PBMC), as estimated by Ro/e. (D) Boxplot comparing the abundances of T/NK cells across adjacent normal (n = 9) and RCC tumor tissues (n = 18). (E) Boxplot comparing the abundances of T/NK cells across ICB sensitive (n = 5) and resistant tumor tissues (n = 4). (F) Dot plot showing the results of KEGG enrichments of the differentially expressed genes of CD8+ T cells in the ICB sensitive and resistant groups. (G) Violin plots comparing the expression of marker genes in the ICB sensitive and resistant tumor cells. The unpaired two-sided Student's t test was used for (D) and (E). Two-sided Wilcoxon test was used for (G).

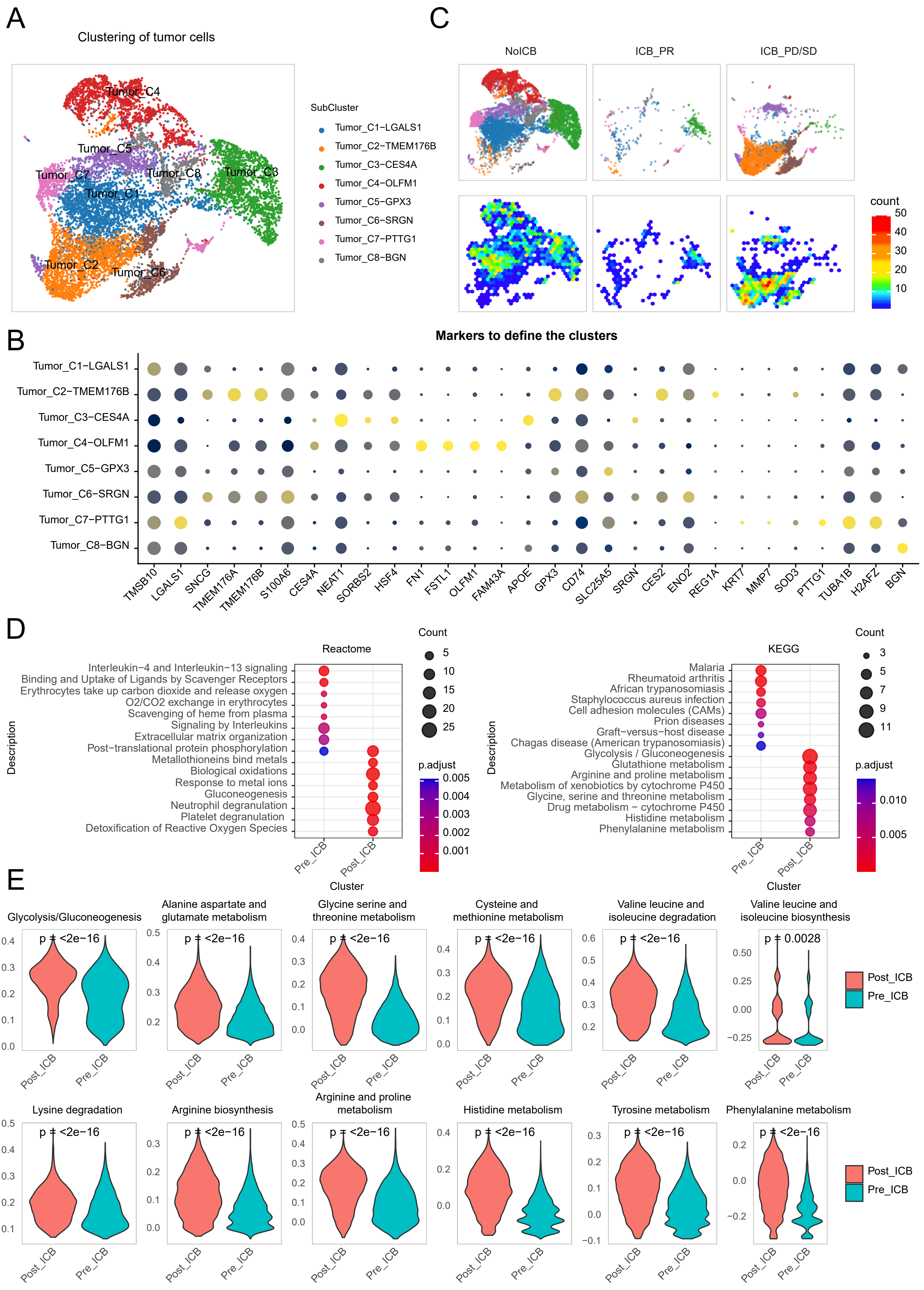
A
Clustering of tumor cells
Tumor_C4
Tumor_C5
Tumor_C7
Tumor_C8
Tumor_C3
Tumor_C1
Tumor_C2
Tumor_C6
SubCluster
Tumor_C1−LGALS1
Tumor_C2−TMEM176B
Tumor_C3−CES4A
Tumor_C4−OLFM1
Tumor_C5−GPX3
Tumor_C6−SRGN
Tumor_C7−PTTG1
Tumor_C8−BGN
C
NoICB
ICB_PR
ICB_PD/SD
count
50
40
30
20
10
B
Markers to define the clusters
Tumor_C1−LGALS1
Tumor_C2−TMEM176B
Tumor_C3−CES4A
Tumor_C4−OLFM1
Tumor_C5−GPX3
Tumor_C6−SRGN
Tumor_C7−PTTG1
Tumor_C8−BGN
TMSB10 LGALS1 SNCG TMEM176A TMEM176B S100A6 CES4A NEAT1 SORBS2 HSF4 FN1 FSTL1 OLFM1 FAM43A APOE GPX3 CD74 SLC25A5 SRGN CES2 ENO2 REG1A KRT7 MMP7 SOD3 PTTG1 TUBA1B H2AFZ BGN
D
Reactome
Interleukin−4 and Interleukin−13 signaling
Binding and Uptake of Ligands by Scavenger Receptors
Erythrocytes take up carbon dioxide and release oxygen
O2/CO2 exchange in erythrocytes
Scavenging of heme from plasma
Signaling by Interleukins
Extracellular matrix organization
Post−translational protein phosphorylation
Metallothioneins bind metals
Biological oxidations
Response to metal ions
Gluconeogenesis
Neutrophil degranulation
Platelet degranulation
Detoxification of Reactive Oxygen Species
Description
Pre_ICB
Post_ICB
Cluster
Count
5
10
15
20
25
p.adjust
0.005
0.004
0.003
0.002
0.001
KEGG
Malaria
Rheumatoid arthritis
African trypanosomiasis
Staphylococcus aureus infection
Cell adhesion molecules (CAMs)
Prion diseases
Graft−versus−host disease
Chagas disease (American trypanosomiasis)
Glycolysis / Gluconeogenesis
Glutathione metabolism
Arginine and proline metabolism
Metabolism of xenobiotics by cytochrome P450
Glycine, serine and threonine metabolism
Drug metabolism − cytochrome P450
Histidine metabolism
Phenylalanine metabolism
Description
Pre_ICB
Post_ICB
Cluster
Count
3
5
7
9
11
p.adjust
0.010
0.005
E
Glycolysis/Gluconeogenesis
p = <2e−16
Alanine aspartate and glutamate metabolism
p = <2e−16
Glycine serine and threonine metabolism
p = <2e−16
Cysteine and methionine metabolism
p = <2e−16
Valine leucine and isoleucine degradation
p = <2e−16
Valine leucine and isoleucine biosynthesis
p = 0.0028
Post_ICB
Pre_ICB
Lysine degradation
p = <2e−16
Arginine biosynthesis
p = <2e−16
Arginine and proline metabolism
p = <2e−16
Histidine metabolism
p = <2e−16
Tyrosine metabolism
p = <2e−16
Phenylalanine metabolism
p = <2e−16
Post_ICB
Pre_ICB

Figure S3. Landscape and metabolic analyses of RCC tumor cells

(A) Uniform Manifold Approximation and Projection (UMAP) embedding of RCC tumor cells. (B) Dot plot showing the markers using to define the clusters of RCC tumor cells. (C) UMAP embedding and cell abundance of scRNA-seq data for tumor cells in immune checkpoint blockade (ICB) treatment-naïve tumor (non-ICB), ICB sensitive tumor (partial response [PR]) and ICB-resistant tumor (progressive or stable diseases [PD/SD]) tissues. (D) Dot plot showing the results of Reactome and KEGG enrichments of the differentially expressed genes in the ICB sensitive and resistant tumor cells. (F) Violin plots comparing the single sample gene set variation analysis (ssGSVA) scores of glycolysis/Gluconeogenesis and amino acid metabolism in the ICB sensitive and resistant tumor cells. Two-sided Wilcoxon test was used for (F).

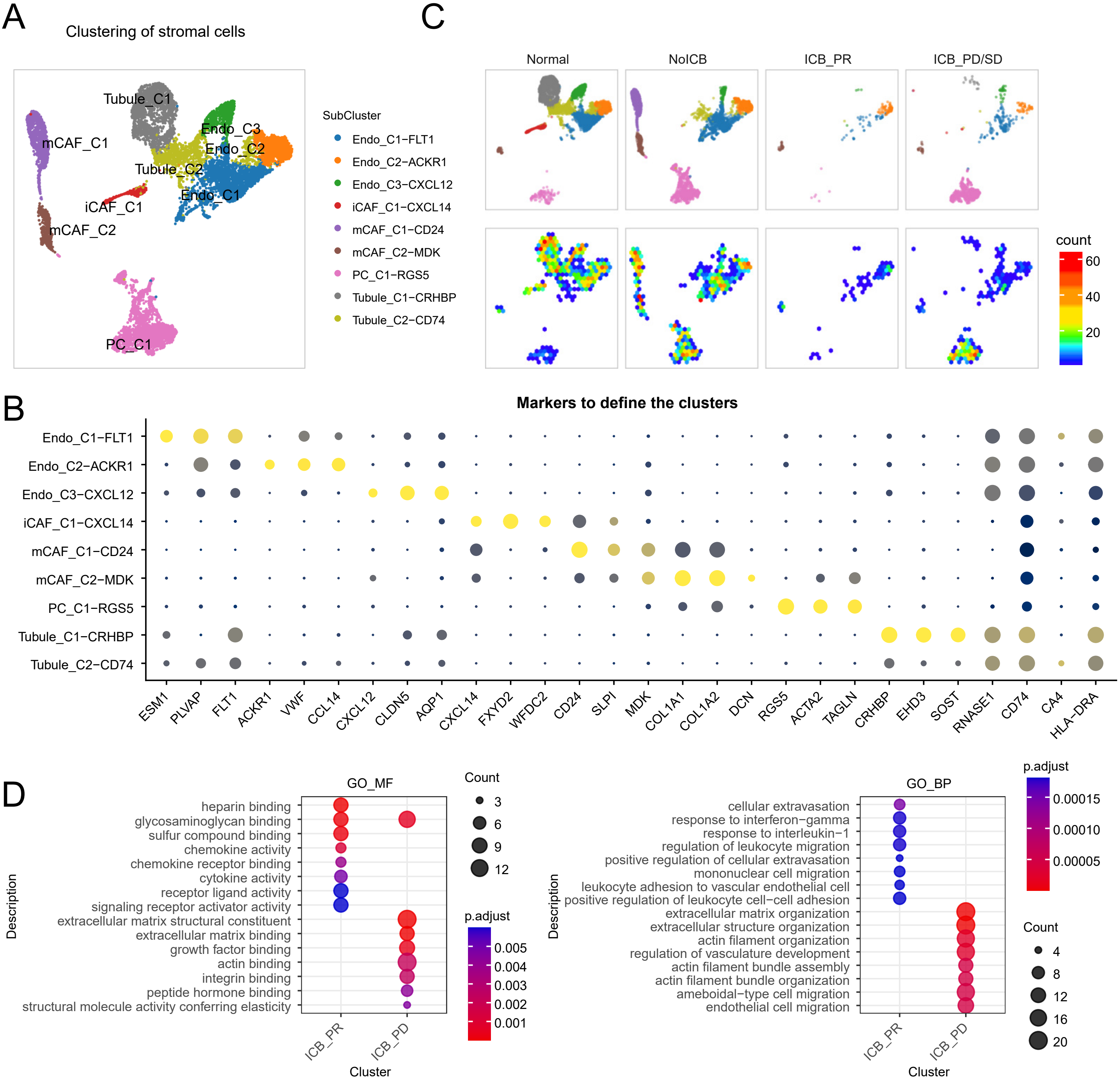

A
Clustering of stromal cells
Tubule_C1
mCAF_C1
Endo_C3
Endo_C2
Tubule_C2
Endo_C1
iCAF_C1
mCAF_C2
PC_C1
SubCluster
Endo_C1−FLT1
Endo_C2−ACKR1
Endo_C3−CXCL12
iCAF_C1−CXCL14
mCAF_C1−CD24
mCAF_C2−MDK
PC_C1−RGS5
Tubule_C1−CRHBP
Tubule_C2−CD74
C
Normal
NoICB
ICB_PR
ICB_PD/SD
count
60
40
20
B
Markers to define the clusters
Endo_C1−FLT1
Endo_C2−ACKR1
Endo_C3−CXCL12
iCAF_C1−CXCL14
mCAF_C1−CD24
mCAF_C2−MDK
PC_C1−RGS5
Tubule_C1−CRHBP
Tubule_C2−CD74
ESM1
PLVAP
FLT1
ACKR1
VWF
CCL14
CXCL12
CLDN5
AQP1
CXCL14
FXYD2
WFDC2
CD24
SLPI
MDK
COL1A1
COL1A2
DCN
RGS5
ACTA2
TAGLN
CRHBP
EHD3
SOST
RNASE1
CD74
CA4
HLA−DRA
D
GO_MF
Description
heparin binding
glycosaminoglycan binding
sulfur compound binding
chemokine activity
chemokine receptor binding
cytokine activity
receptor ligand activity
signaling receptor activator activity
extracellular matrix structural constituent
extracellular matrix binding
growth factor binding
actin binding
integrin binding
peptide hormone binding
structural molecule activity conferring elasticity
ICB_PR
ICB_PD
Cluster
Count
3
6
9
12
p.adjust
0.005
0.004
0.003
0.002
0.001
GO_BP
Description
cellular extravasation
response to interferon−gamma
response to interleukin−1
regulation of leukocyte migration
positive regulation of cellular extravasation
mononuclear cell migration
leukocyte adhesion to vascular endothelial cell
positive regulation of leukocyte cell−cell adhesion
extracellular matrix organization
extracellular structure organization
actin filament organization
regulation of vasculature development
actin filament bundle assembly
actin filament bundle organization
ameboidal−type cell migration
endothelial cell migration
ICB_PR
ICB_PD
Cluster
p.adjust
0.00015
0.00010
0.00005
Count
4
8
12
16
20

Figure S4. Landscape of RCC stromal cells

(A) Uniform Manifold Approximation and Projection (UMAP) embedding of RCC stromal cells. (B) Dot plot showing the markers using to define the clusters of RCC stromal cells. (C) UMAP embedding and cell abundance of scRNA-seq data for stromal cells in adjacent normal, immune checkpoint blockade (ICB) treatment-naïve tumor (non-ICB), ICB sensitive tumor (partial response [PR]) and ICB-resistant tumor (progressive or stable diseases [PD/SD]) tissues. (D) Dot plot showing the results of GO BP (biological process) and MF (molecular function) enrichments of the differentially expressed genes in the ICB PR and ICB PD/SD stromal cells.

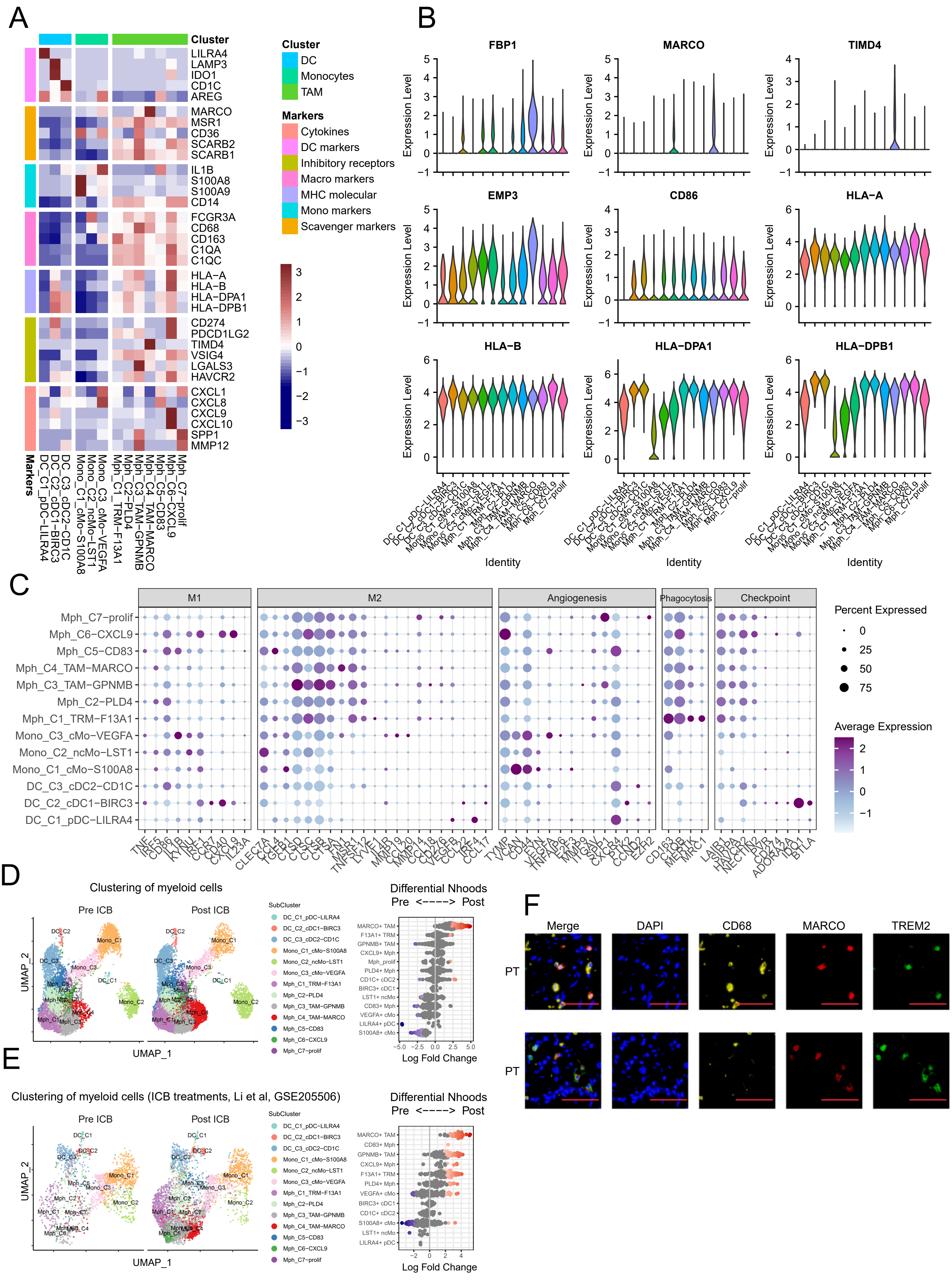
A
Cluster
LILRA4
LAMP3
IDO1
CD1C
AREG
MARCO
MSR1
CD36
SCARB2
SCARB1
IL1B
S100A8
S100A9
CD14
FCGR3A
CD68
CD163
C1QA
C1QC
HLA-A
HLA-B
HLA-DPA1
HLA-DPB1
CD274
PDCD1LG2
TIMD4
VSIG4
LGALS3
HAVCR2
CXCL1
CXCL8
CXCL9
CXCL10
SPP1
MMP12
Markers
DC_C1_pDC-LILRA4
DC_C2_cDC1-BIRC3
DC_C3_cDC2-CD1C
Mono_C1_cMo-S100A8
Mono_C2_ncMo-LST1
Mono_C3_cMo-VEGFA
Mph_C1_TRM-F13A1
Mph_C2-PLD4
Mph_C3_TAM-GPNMB
Mph_C4_TAM-MARCO
Mph_C5-CD83
Mph_C6-CXCL9
Mph_C7-prolif
Cluster
DC
Monocytes
TAM
Markers
Cytokines
DC markers
Inhibitory receptors
Macro markers
MHC molecular
Mono markers
Scavenger markers
3
2
1
0
−1
−2
−3
B
FBP1
MARCO
TIMD4
EMP3
CD86
HLA−A
HLA−B
HLA−DPA1
HLA−DPB1
Expression Level
Identity
C
M1
M2
Angiogenesis
Phagocytosis
Checkpoint
Mph_C7−prolif
Mph_C6−CXCL9
Mph_C5−CD83
Mph_C4_TAM−MARCO
Mph_C3_TAM−GPNMB
Mph_C2−PLD4
Mph_C1_TRM−F13A1
Mono_C3_cMo−VEGFA
Mono_C2_ncMo−LST1
Mono_C1_cMo−S100A8
DC_C3_cDC2−CD1C
DC_C2_cDC1−BIRC3
DC_C1_pDC−LILRA4
Percent Expressed
0
25
50
75
Average Expression
2
1
0
−1
TNF IRF5 CD86 IL1B KYNU IRF1 CCR7 CD40 CXCL9 IL23A
CLEC7A CCL4 TGFB1 CTSD CTSC CTSB CTSA FN1 MSR1 TNFSF12 LYVE1 IL4R MMP19 CCL20 MMP14 CCL18 CD276 VEGFB CCL22 IRF4 CCL17
TYMP VCAN CD44 VEGFA TNFAIP6 F3 MMP9 ITGAV SPP1 CXCR4 PTK2 CCND2 EZH2
CD163 C1QB MERTK MRC1
LAIR1 SIRPA HAVCR2 NECTIN2 PVR CD274 ADORA2A IDO1 BTLA
D
Clustering of myeloid cells
Pre ICB
Post ICB
UMAP_1
UMAP_2
SubCluster
DC_C1_pDC−LILRA4
DC_C2_cDC1−BIRC3
DC_C3_cDC2−CD1C
Mono_C1_cMo−S100A8
Mono_C2_ncMo−LST1
Mono_C3_cMo−VEGFA
Mph_C1_TRM−F13A1
Mph_C2−PLD4
Mph_C3_TAM−GPNMB
Mph_C4_TAM−MARCO
Mph_C5−CD83
Mph_C6−CXCL9
Mph_C7−prolif
Differential Nhoods
Pre <----> Post
MARCO+ TAM
F13A1+ TRM
GPNMB+ TAM
CXCL9+ Mph
Mph_prolif
PLD4+ Mph
CD1C+ cDC2
BIRC3+ cDC1
LST1+ ncMo
CD83+ Mph
VEGFA+ cMo
LILRA4+ pDC
S100A8+ cMo
Log Fold Change
E
Clustering of myeloid cells (ICB treatments, Li et al, GSE205506)
Pre ICB
Post ICB
UMAP_1
UMAP_2
SubCluster
Differential Nhoods
Pre <----> Post
MARCO+ TAM
CD83+ Mph
GPNMB+ TAM
CXCL9+ Mph
F13A1+ TRM
PLD4+ Mph
VEGFA+ cMo
BIRC3+ cDC1
CD1C+ cDC2
S100A8+ cMo
LST1+ ncMo
LILRA4+ pDC
Log Fold Change
F
Merge
DAPI
CD68
MARCO
TREM2
PT
PT

Figure S5. Markers to define the cell lineages of myeloid cells

(A) Heatmap showing the markers using to define the myeloid cell clusters. (B) Dot plot showing the markers using to define the myeloid cell clusters. (C) Dot plot showing the markers of M1 macrophage, M2 macrophage, angiogenesis, phagocytosis and checkpoints in the myeloid cell clusters. (D) Comparison of the abundance of myeloid cell clusters in the pre-ICB (n = 9) and post-ICB samples (n = 9) in the RCC single-cell atlas. (E) Comparison of the abundance of myeloid cell clusters in the pre-ICB (n = 10) and post-ICB samples (n = 16) in an external cohort. The data was derived from colorectal cancer (GSE205506) reported by Li et al. The cell annotation was transferred from the RCC single-cell atlas. (F) Representative images of multiplex immunofluorescence staining of DAPI, CD68, MARCO, TREM2 and TGFB in RCC tumors. Representative experiment out of n = 3 biological replicates. Scale bar, 50 μm.

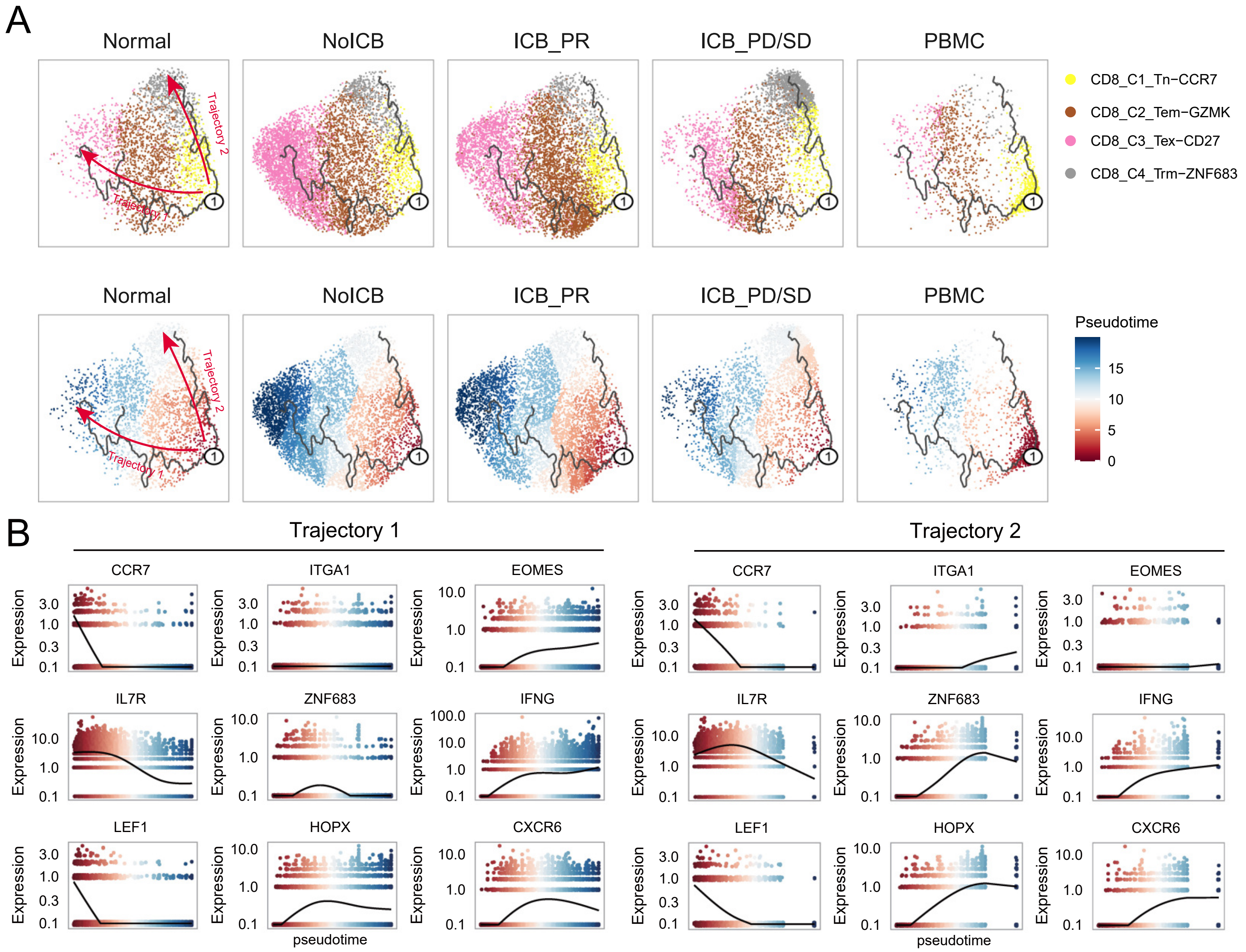
A
Normal
NoICB
ICB_PR
ICB_PD/SD
PBMC
Trajectory 1
Trajectory 2
CD8_C1_Tn−CCR7
CD8_C2_Tem−GZMK
CD8_C3_Tex−CD27
CD8_C4_Trm−ZNF683
Pseudotime
B
Trajectory 1
Trajectory 2
CCR7
ITGA1
EOMES
IL7R
ZNF683
IFNG
LEF1
HOPX
CXCR6
Expression
pseudotime

Figure S6. Cell trajectory of CD8+ T cells

(A) Cell trajectory and pseudotime of CD8+ T cells for adjacent normal, immune checkpoint blockade (ICB) treatment-naïve tumor (non-ICB), ICB sensitive tumor (partial response [PR]), ICB-resistant tumor (progressive or stable diseases [PD/SD]) and peripheral blood mononuclear cell (PBMC) tissues. (B) Expression of marker genes of CD8+ T cells by pseudotime.

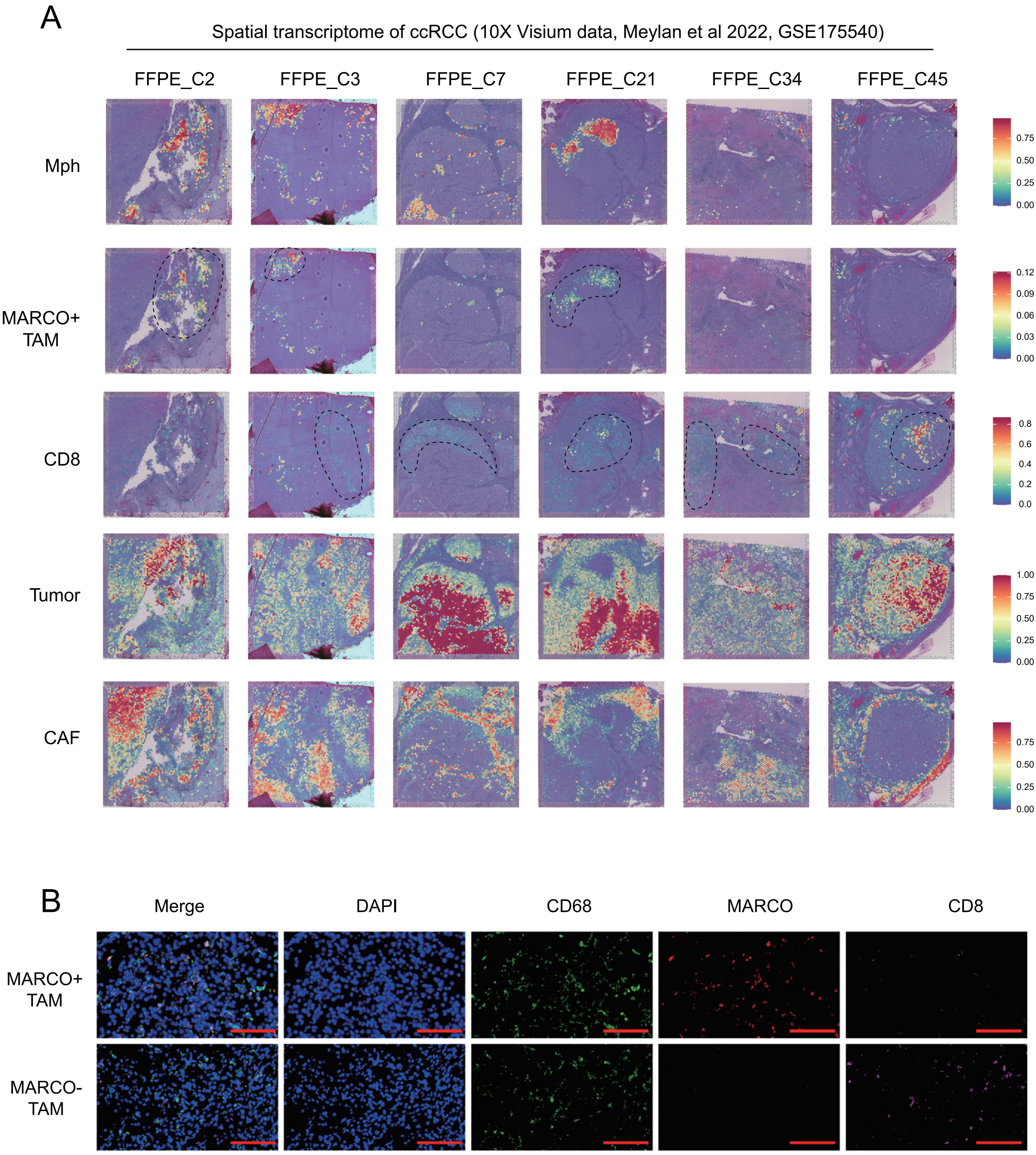
A
Spatial transcriptome of ccRCC (10X Visium data, Meylan et al 2022, GSE175540)
FFPE_C2
FFPE_C3
FFPE_C7
FFPE_C21
FFPE_C34
FFPE_C45
Mph
MARCO+ TAM
CD8
Tumor
CAF
0.75
0.50
0.25
0.00
0.12
0.09
0.06
0.03
0.00
0.8
0.6
0.4
0.2
0.0
1.00
0.75
0.50
0.25
0.00
0.75
0.50
0.25
0.00
B
Merge
DAPI
CD68
MARCO
CD8
MARCO+ TAM
MARCO- TAM

Figure S7. ***In situ*** **interaction analyses of MARCO+ TAM and CD8+ T cells**

(A) *In situ* interaction analyses of MARCO+ TAM and CD8+ T cells using spatial transcriptome of ccRCC (10X Visium data, Meylan et al 2022, GSE175540). The cell-lineage specific scores were transferred from the RCC single-cell atlas. (B) Representative images of mIF comparing in situ co-localization of CD8+ T cells and MARCO+ TAMs in RCC samples. Four markers were stained: DAPI, CD68, MARCO and CD8. Representative experiment out of n = 3 biological replicates. Scale bar, 100 µm.

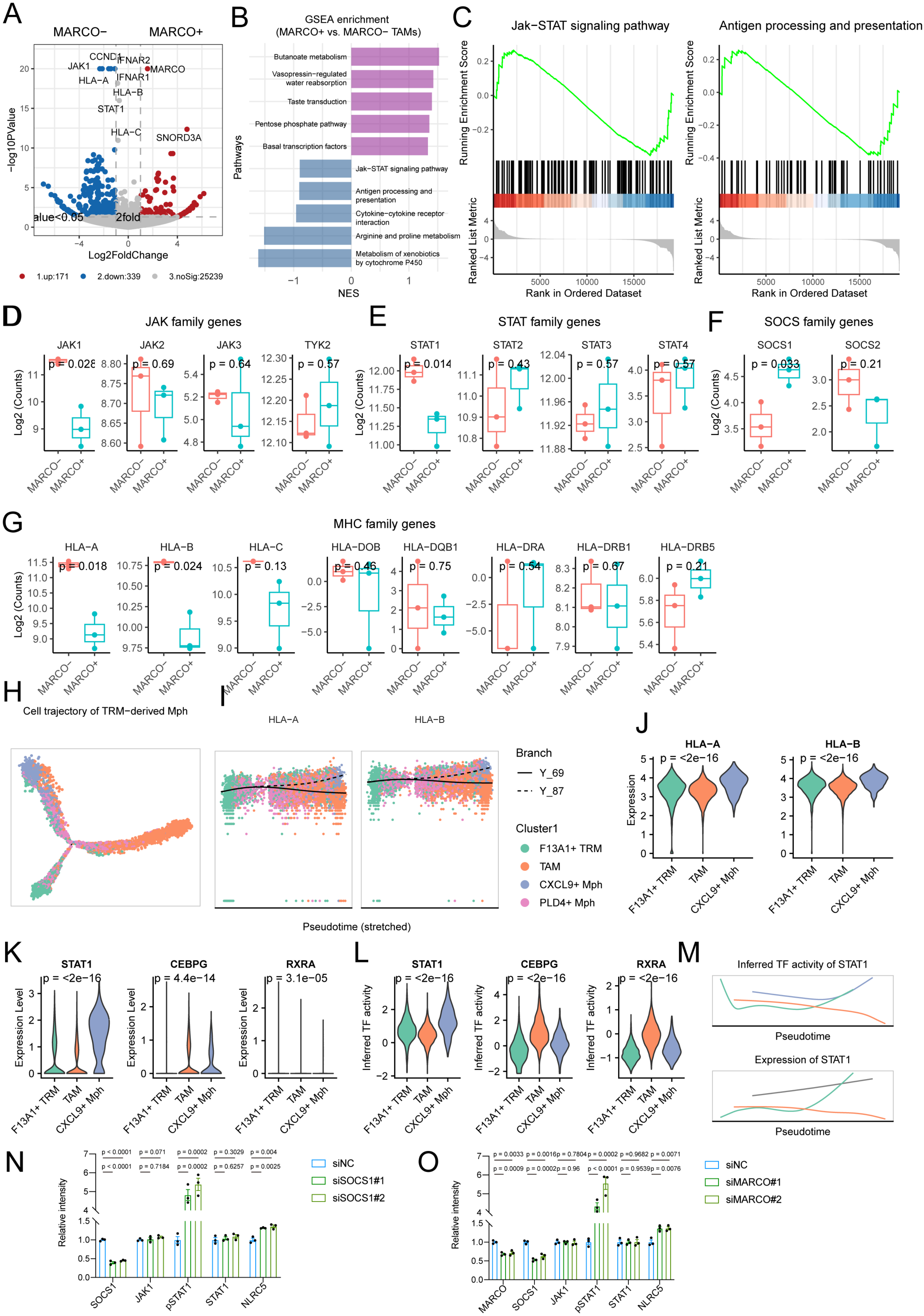
A
MARCO−
MARCO+
CCND1
IFNAR2
JAK1
MARCO
HLA−A
IFNAR1
HLA−B
STAT1
HLA−C
SNORD3A
−log10PValue
Log2FoldChange
alue<0.05
2fold
1.up:171
2.down:339
3.noSig:25239
B
GSEA enrichment
(MARCO+ vs. MARCO− TAMs)
Butanoate metabolism
Vasopressin−regulated water reabsorption
Taste transduction
Pentose phosphate pathway
Basal transcription factors
Jak−STAT signaling pathway
Antigen processing and presentation
Cytokine−cytokine receptor interaction
Arginine and proline metabolism
Metabolism of xenobiotics by cytochrome P450
Pathways
NES
C
Jak−STAT signaling pathway
Antigen processing and presentation
Running Enrichment Score
Ranked List Metric
Rank in Ordered Dataset
D
JAK family genes
JAK1
JAK2
JAK3
TYK2
Log2 (Counts)
p = 0.028
p = 0.69
p = 0.64
p = 0.57
E
STAT family genes
STAT1
STAT2
STAT3
STAT4
p = 0.014
p = 0.43
p = 0.57
p = 0.57
F
SOCS family genes
SOCS1
SOCS2
p = 0.033
p = 0.21
G
MHC family genes
HLA−A
HLA−B
HLA−C
HLA−DOB
HLA−DQB1
HLA−DRA
HLA−DRB1
HLA−DRB5
p = 0.018
p = 0.024
p = 0.13
p = 0.46
p = 0.75
p = 0.54
p = 0.67
p = 0.21
H
Cell trajectory of TRM−derived Mph
I
HLA−A
HLA−B
Pseudotime (stretched)
Branch
Y_69
Y_87
Cluster1
F13A1+ TRM
TAM
CXCL9+ Mph
PLD4+ Mph
J
HLA−A
HLA−B
p = <2e−16
Expression
K
STAT1
CEBPG
RXRA
p = <2e−16
p = 4.4e−14
p = 3.1e−05
Expression Level
L
STAT1
CEBPG
RXRA
Inferred TF activity
M
Inferred TF activity of STAT1
Pseudotime
Expression of STAT1
Pseudotime
N
Relative intensity
SOCS1
JAK1
pSTAT1
STAT1
NLRC5
siNC
siSOCS1#1
siSOCS1#2
p < 0.0001
p = 0.071
p = 0.0002
p = 0.3029
p = 0.004
p < 0.0001
p = 0.7184
p = 0.0002
p = 0.6257
p = 0.0025
O
Relative intensity
MARCO
SOCS1
JAK1
pSTAT1
STAT1
NLRC5
siNC
siMARCO#1
siMARCO#2
p = 0.0033
p = 0.0016
p = 0.7804
p = 0.0002
p = 0.9682
p = 0.0071
p = 0.0009
p = 0.0002
p = 0.96
p < 0.0001
p = 0.9539
p = 0.0076

Figure S8. Validation of the MARCO-SOCS1-JAK1-STAT1 pathway using human RCC-derived MARCO+ TAM and MARCO- TAM

(A) Transcriptome sequencing was performed on MARCO+ TAMs (n = 3) and MARCO- TAMs (n = 3) from human RCC samples. (B) Top 5 pathways changed in MARCO+ TAMs (n = 3) and MARCO- TAMs (n = 3) revealed by KEGG gene-set enrichment analysis (GSEA) pathway enrichment analysis. (C) GSEA results showed that JAK-STAT pathway and antigen presentation pathway were down-regulated in MARCO+ TAMs. (D) Boxplot to compare the expression of JAK family genes from MARCO+ TAMs (n = 3) and MARCO- TAMs (n = 3). (E) Boxplot to compare the expression of STAT family genes from MARCO+ TAMs (n = 3) and MARCO- TAMs (n = 3). (F) Boxplot to compare the expression of SOCS family genes from MARCO+ TAMs (n = 3) and MARCO- TAMs (n = 3). (G) Boxplot to compare the expression of MHC family genes from MARCO+ TAMs (n = 3) and MARCO- TAMs (n = 3). (H) Pseudotime and cell trajectories of tissue-resident macrophages (TRM)-derived macrophages inferred by Monocle2. (I) Expression of HLA-A and HLA-B by pseudotime in cell trajectories of TRM-derived macrophages. (J) Expression of HLA-A and HLA-B in cell trajectories of TRM-derived macrophages. (K) Expression and (L) transcription factor activity of STAT1, CEBPG and RXRA in cell trajectories of TRM-derived macrophages. (M) Dynamic changes in transcription factor activity (top) and expression (bottom) of STAT1 over time. (N) The quantitation of band intensity of SOCS1, JAK1, STAT1, p-STAT1 and NLRC5 in TAMs from siNC and siSOCS1 groups using β-Actin as loading control (n = 3). Data are presented as mean ± SEM. (O) The quantitation of band intensity of MARCO, SOCS1, JAK1, STAT1, p-STAT1 and NLRC5 in TAMs from siNC and siMARCO groups using β-Actin as loading control (n = 3). Data are presented as mean ± SEM. The unpaired two-sided Student's t test was used for (D) (E) (F) (G) (N) and (O). Two-sided Kruskal-Wallis test was used for (J) (K) and (L).

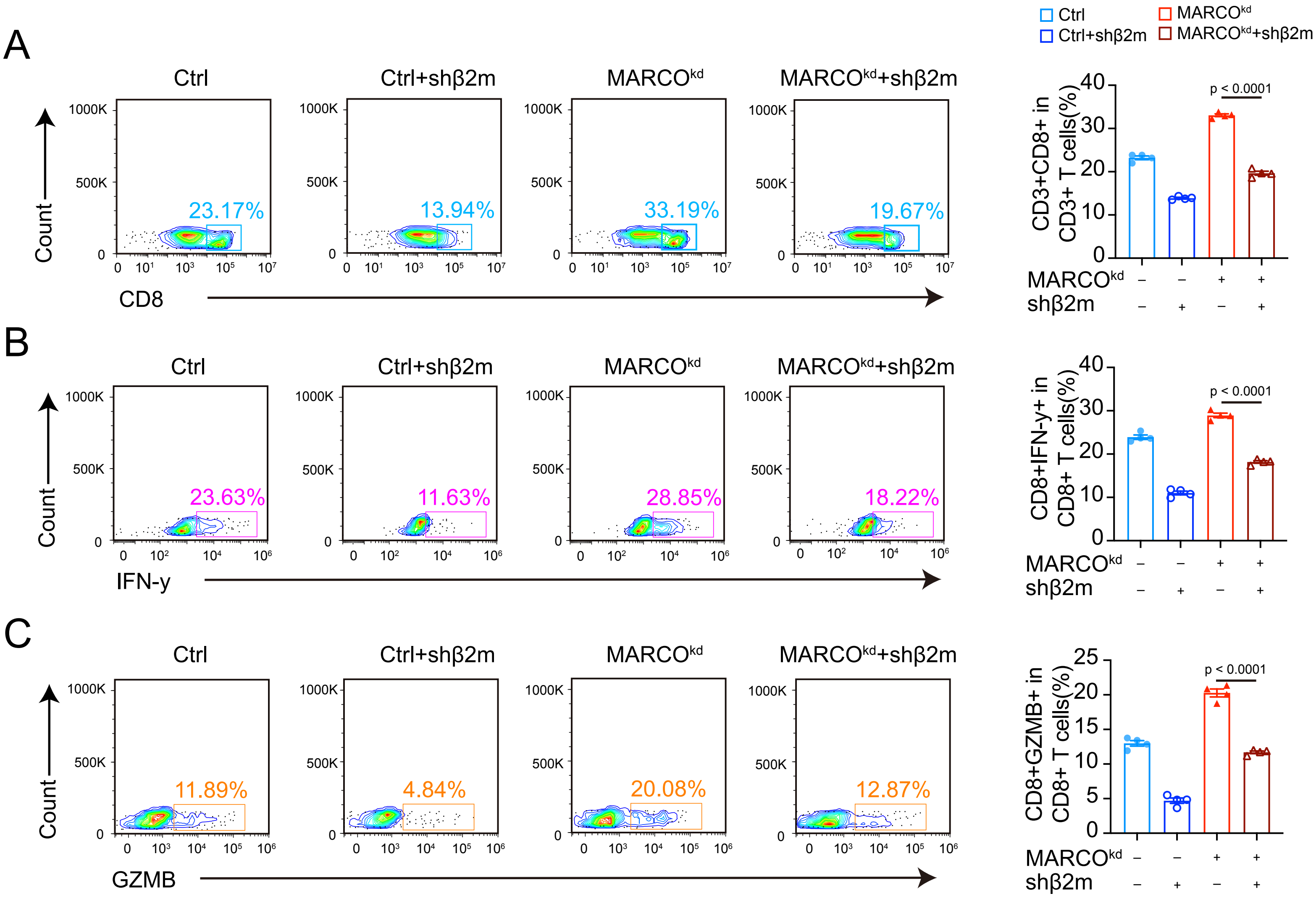

A
B
C
Ctrl
Ctrl+shβ2m
MARCOkd
MARCOkd+shβ2m
Count
CD8
IFN-y
GZMB
23.17%
13.94%
33.19%
19.67%
23.63%
11.63%
28.85%
18.22%
11.89%
4.84%
20.08%
12.87%
CD3+CD8+ in CD3+ T cells(%)
CD8+IFN-y+ in CD8+ T cells(%)
CD8+GZMB+ in CD8+ T cells(%)
p < 0.0001
MARCOkd
shβ2m

Figure S9. Representative contour plot of tumor-infiltrating (A) CD8+ T cells, (B) IFN-γ+ CD8+ T cells and (C) GZMB+ CD8+ T cells in subcutaneous RCC tumor samples as determined by flow cytometric analysis. Data are presented as mean ± SEM. The unpaired two-sided Student's t test was used for (A) (B) and (C).

A

Enriched TFs in the ATAC-seq peaks around SOCS1 (mouse)

Rank	Motif	p-Value	Motif name	TF
1	GGCCGTGGCAGC	1e-19	SP3/MA0746.3/Jaspar(0.666)	SP3
2	CCGATTTCTAAG	1e-18	Hmga1/MA2124.1/Jaspar(0.645)	HMGA1
3	ACCAACTCGTGG	1e-17	ZNF707/MA1715.1/Jaspar(0.729)	ZNF707
4	TGACTCATGCTA	1e-14	Jun/MA0489.3/Jaspar(0.776)	JUN
5	TGAATGAAGGCG	1e-14	PB0028.1_Hbp1_1/Jaspar(0.740)	HBP1
6	GCAGTCAAAATG	1e-13	Yy1/MA0095.4/Jaspar(0.697)	YY1
7	CTGCTAACCACT	1e-12	PH0168.1_Hnf1b/Jaspar(0.672)	HNF1B
8	TGCACTTCCC	1e-12	PU.1(ETS)/ThioMac-PU.1-ChIP-Seq(GSE21512)/Homer(0.865)	**SPI1**
9	CGCACACATC	1e-12	PB0208.1_Zscan4_2/Jaspar(0.719)	ZSCAN4
10	CCCTGGAATTGC	1e-12	ZNF530/MA1981.2/Jaspar(0.652)	ZNF530

B

Enriched TFs for SOCS1 (hTFtarget database, human)

TF	Tissue	No. of peaks (total/average)	Peaks in gene body (total/average)	Peaks around TSS (total/average)
**SPI1**	**Adrenal gland**	**13/4**	**5/1**	**8/2**
IRF2	Adult HSPCs	2/2	1/1	1/1
CTCF	Aortic adventitial	13/6	6/3	7/3
MYC	Ascitic fluid	1/1	1/1	0/0
BATF	Blood	8/4	3/1	5/2
BC11A	Blood	5/5	2/2	3/3
BCL3	Blood	2/2	1/1	1/1
BCL6	Blood	4/4	3/3	1/1
BHLHE40	Blood	1/1	1/1	0/0
BRD3	Blood	4/2	2/1	2/1

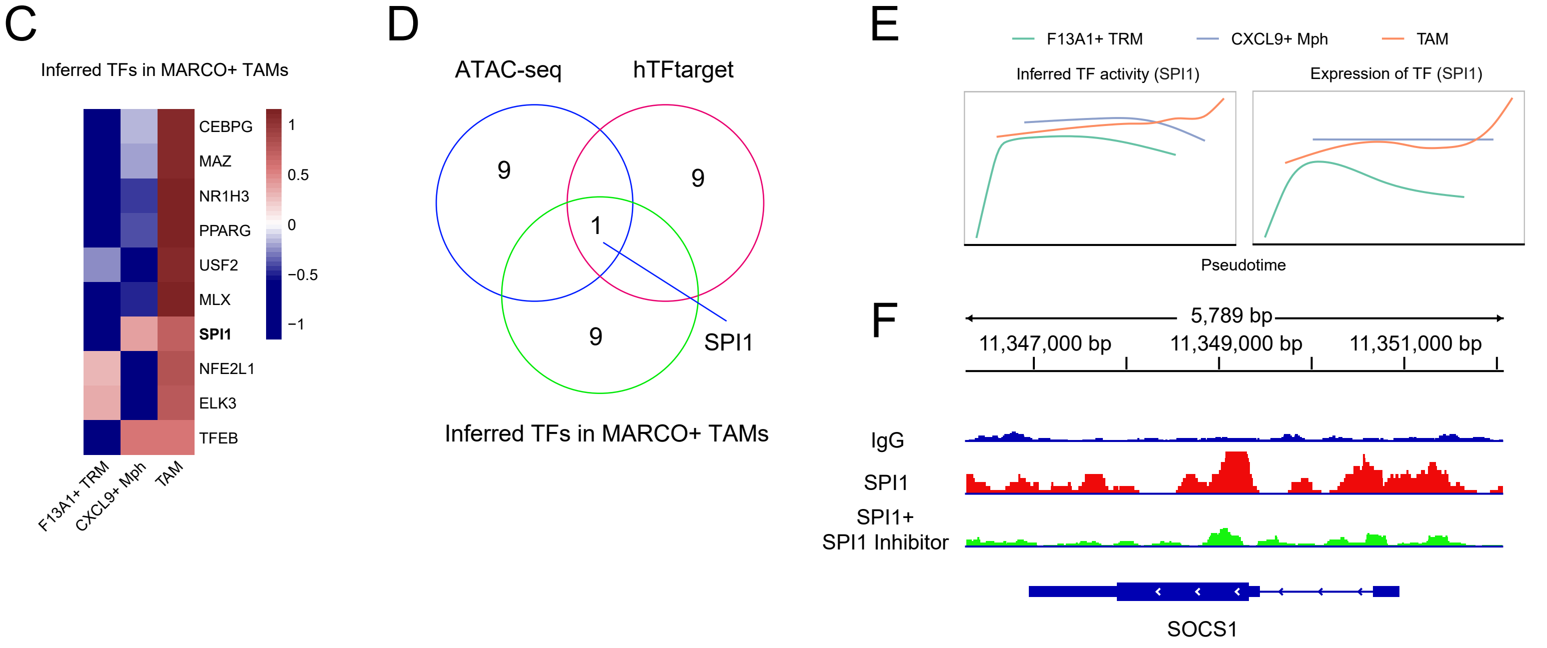

Figure S10. ATAC-seq data to investigate the upstream transcript factors mediated by MARCO to regulate SOCS1

(A) Top 10 enriched transcript factors (TFs) in the ATAC-seq peaks detected in siNC TAMs compared by siMARCO TAMs. (B) Top 10 enriched TFs for the target gene (SCOS1) in the hTFtarget databases. (C) Top 10 up-regulated TFs according to transcription factor activity in MARCO+ TAMs. (D) Intersection of the three datasets to investigate the upstream transcript factors mediated by MARCO to regulate SOCS1. (E) Dynamic changes in transcription factor activity (left) and expression (right) of SPI1 over time. (F) The ATAC-seq peaks detected in IgG, SPI1 and SPI1+SPI1 inhibitor groups. The data was derived from the GEO database (GSE236085).

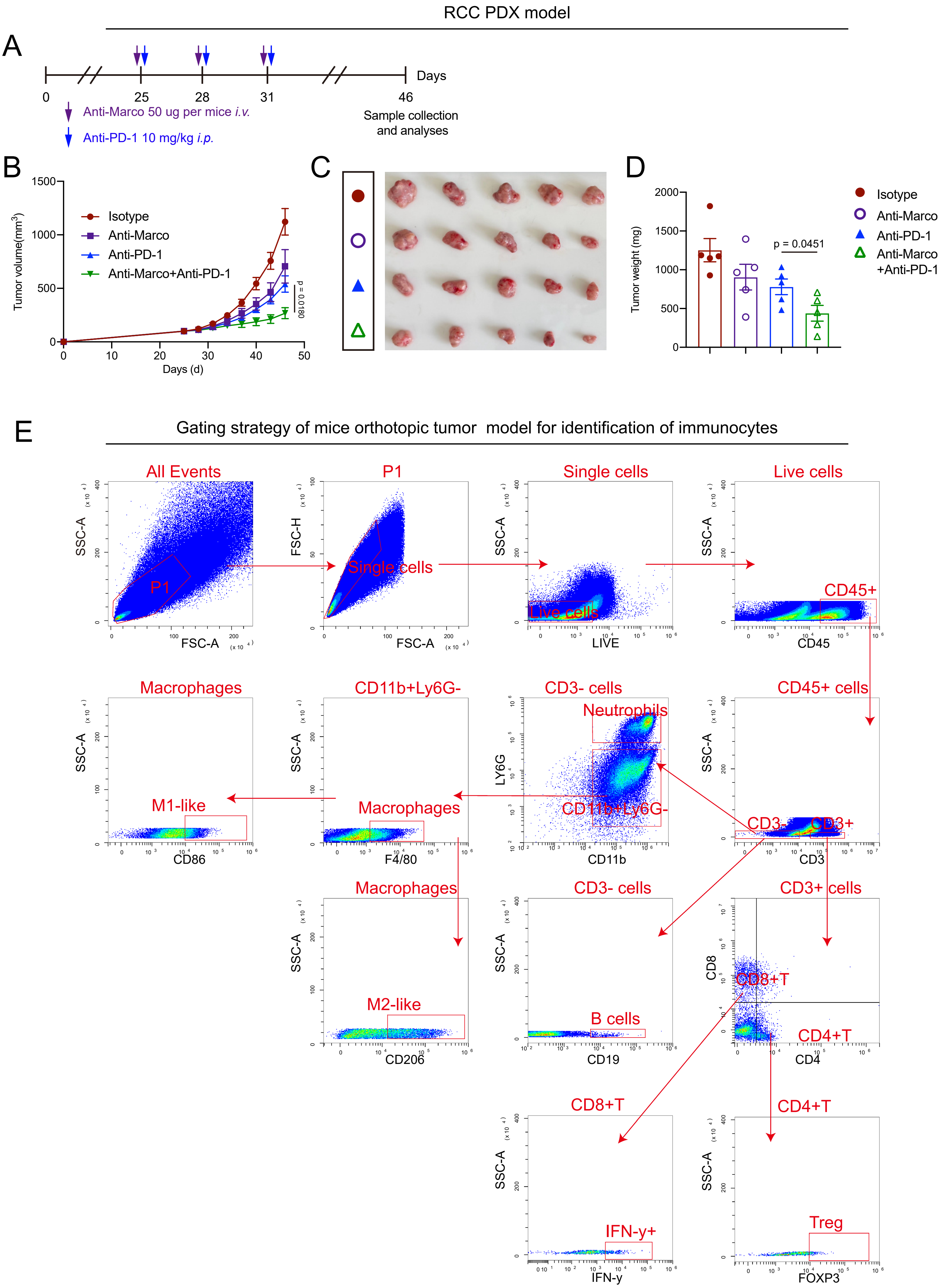
RCC PDX model
A
Days
0
25
28
31
46
Anti-Marco 50 ug per mice i.v.
Anti-PD-1 10 mg/kg i.p.
Sample collection and analyses
B
Tumor volume(mm3)
Days (d)
Isotype
Anti-Marco
Anti-PD-1
Anti-Marco+Anti-PD-1
p = 0.0180
C
D
Tumor weight (mg)
p = 0.0451
Isotype
Anti-Marco
Anti-PD-1
Anti-Marco +Anti-PD-1
E
Gating strategy of mice orthotopic tumor model for identification of immunocytes
All Events
P1
Single cells
Live cells
CD45+
CD45+ cells
CD3- cells
Neutrophils
CD11b+Ly6G-
CD3-
CD3+
Macrophages
M1-like
M2-like
B cells
CD3+ cells
CD8+T
CD4+T
IFN-y+
Treg
SSC-A
FSC-A
FSC-H
LIVE
CD45
CD3
LY6G
CD11b
F4/80
CD86
CD206
CD19
CD8
CD4
IFN-y
FOXP3

Figure S11. Anti-tumor effect of MARCO blockade and anti-PD-1 combination therapies in RCC PDX model, and gating strategy of mice orthotopic tumor model

(A) Flow chart of the experimental design. The mice were treated with either anti-MARCO antibody (50 μg per mouse) with or without anti-PD-1 antibody (10 mg/kg body weight) as indicated starting from day 25. (B) Growth curves of tumors in the different treatment groups. n = 5 per group. Data are presented as mean ± SEM. (C) Tumor image of tumors in the different treatment groups. n = 5 per group. (D) Tumor weight of mice in the different treatment groups. n = 5 per group. Data are presented as mean ± SEM. (E) Gating strategy for identification of macrophages, M1-like macrophages, M2-like macrophages, NK cells, neutrophils, CD45+ leukocytes, CD3+ T cells, CD8+ T cells, IFN-γ+CD8+ T cells, CD4+ T cells and B cells. The unpaired two-sided Student's t test was used for (B) and (D).

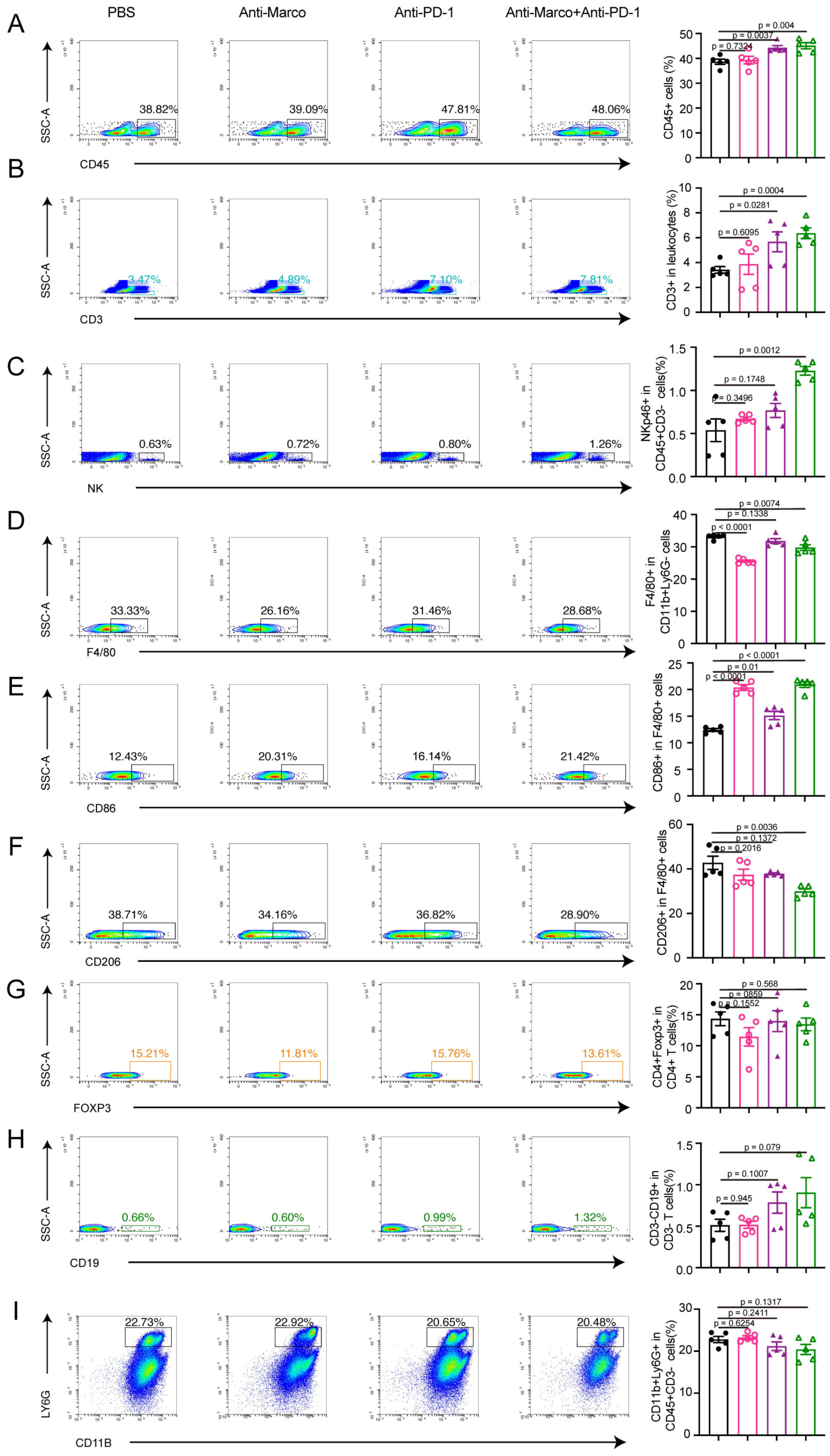

PBS
Anti-Marco
Anti-PD-1
Anti-Marco+Anti-PD-1
A
38.82%
39.09%
47.81%
48.06%
SSC-A
CD45
CD45+ cells (%)
p = 0.7324
p = 0.0037
p = 0.004
B
3.47%
4.89%
7.10%
7.81%
CD3
CD3+ in leukocytes (%)
p = 0.6095
p = 0.0281
p = 0.0004
C
0.63%
0.72%
0.80%
1.26%
NK
NKp46+ in CD45+CD3- cells(%)
p = 0.3496
p = 0.1748
p = 0.0012
D
33.33%
26.16%
31.46%
28.68%
F4/80
F4/80+ in CD11b+Ly6G- cells
p < 0.0001
p = 0.1338
p = 0.0074
E
12.43%
20.31%
16.14%
21.42%
CD86
CD86+ in F4/80+ cells
p < 0.0001
p = 0.01
p < 0.0001
F
38.71%
34.16%
36.82%
28.90%
CD206
CD206+ in F4/80+ cells
p = 0.2016
p = 0.1372
p = 0.0036
G
15.21%
11.81%
15.76%
13.61%
FOXP3
CD4+Foxp3+ in CD4+ T cells(%)
p = 0.1552
p = 0859
p = 0.568
H
0.66%
0.60%
0.99%
1.32%
CD19
CD3-CD19+ in CD3- T cells(%)
p = 0.945
p = 0.1007
p = 0.079
I
22.73%
22.92%
20.65%
20.48%
LY6G
CD11B
CD11b+Ly6G+ in CD45+CD3- cells(%)
p = 0.6254
p = 0.2411
p = 0.1317

Figure S12. Representative flow cytometry plots and statistical analysis of the ratios of immunocytes within orthotopic Renca tumors from mice in different treatment groups on day 21

Tumor-infiltrating immunocytes include (A) CD45+ leukocytes, (B) CD3+ T cells, (C) NK cells, (D) macrophages, (E) M1-like macrophages, (F) M2-like macrophages, (G) Tregs, (H) B cells, and (I) neutrophils. n = 5 in each group. Data are presented as mean ± SEM. The unpaired two-sided Student's t test was used for (A) (B) (C) (D) (E) (F) (G) (H) and (I).
